# Genome-wide Identification of Tebufenozide Resistant Genes in the smaller tea tortrix, *Adoxophyes honmai* (Lepidoptera: Tortricidae)

**DOI:** 10.1038/s41598-019-40863-5

**Published:** 2019-03-12

**Authors:** Miwa Uchibori-Asano, Akiya Jouraku, Toru Uchiyama, Kakeru Yokoi, Gaku Akiduki, Yoshitaka Suetsugu, Tetsuya Kobayashi, Akihito Ozawa, Saki Minami, Chiharu Ishizuka, Yoshiaki Nakagawa, Takaaki Daimon, Tetsuro Shinoda

**Affiliations:** 1 Institute of Agrobiological Sciences, National Agriculture and Food Research Organization (NARO), Tsukuba, Ibaraki 305-8634 Japan; 2 Tea Research Center, Shizuoka Research Institute of Agriculture and Forestry, Kurasawa, Kikugawa, Shizuoka 439-0002 Japan; 3 Kyushu Okinawa Agricultural Research Center, National Agriculture and Food Research Organization (NARO), Kumamoto, 861-1192 Japan; 4 Division of Applied Life Sciences, Graduate School of Agriculture, Kyoto University, Kitashirakawa Oiwake-cho, Sakyo-ku, Kyoto 606-8502 Japan; 5 Department of Applied Biosciences, Graduate School of Agriculture, Kyoto University, Kitashirakawa Oiwake-cho, Sakyo-ku, Kyoto 606-8502 Japan

## Abstract

The smaller tea tortrix, *Adoxophyes honmai*, has developed strong resistance to tebufenozide, a diacylhydrazine-type (DAH) insecticide. Here, we investigated its mechanism by identifying genes responsible for the tebufenozide resistance using various next generation sequencing techniques. First, double-digest restriction site-associated DNA sequencing (ddRAD-seq) identified two candidate loci. Then, synteny analyses using *A. honmai* draft genome sequences revealed that one locus contained the ecdysone receptor gene (*EcR*) and the other multiple *CYP9A* subfamily P450 genes. RNA-seq and direct sequencing of *EcR* cDNAs found a single nucleotide polymorphism (SNP), which was tightly linked to tebufenozide resistance and generated an amino acid substitution in the ligand-binding domain. The binding affinity to tebufenozide was about 4 times lower in *in vitro* translated EcR of the resistant strain than in the susceptible strain. RNA-seq analyses identified commonly up-regulated genes in resistant strains, including *CYP9A* and choline/carboxylesterase (*CCE*) genes. RT-qPCR analysis and bioassays showed that the expression levels of several *CYP9A* and *CCE* genes were moderately correlated with tebufenozide resistance. Collectively, these results suggest that the reduced binding affinity of EcR is the main factor and the enhanced detoxification activity by some CYP9As and CCEs plays a supplementary role in tebufenozide resistance in *A. honmai*.

## Introduction

Diacylhydrazine-type insect growth regulators (DAH-IGRs) are non-steroidal ecdysteroid agonists and tebufenozide, methoxyfenozide, chromafenozide, and halofenozide are representatives of commercially available DAH-IGRs^1^. The former three DAH-IGRs are specific to lepidopteran pests, while halofenozide is effective to both lepidopteran and coleopteran pests. Due to their low toxicity to non-target organisms, including natural enemies and pollinators, DAH-IGRs are recognized as environment-friendly insecticides, and therefore highly favorable for crop protection in modern agricultural systems.

The target molecule of DAH-IGRs is the product of the *ecdysone receptor* (*EcR*) gene, which forms a functional ecdysteroid receptor complex with the partner protein ultraspiracle (USP)^2^. Ecdysone, the precursor of the main active compound of ecdysteroids, i.e., 20-hydroxyecdysone (20E), is secreted periodically from the prothoracic gland into the haemolymph during insect development. The secreted ecdysone is converted to 20E in the peripheral tissues, and this 20E binds the EcR present in target tissues such as the epidermis, and initiates the moulting and metamorphosis processes via activating the ecdysteroid-signalling cascade, ultimately leading to the shed off of old cuticles^3^. DAH-IGRs also induce these processes via binding the EcR but at inappropriate developmental timing. Eventually, DAH-IGRs force the induction of moulting processes, such as cessation of feeding and head-capsule slippage at unnatural timing, and lead to lethal moulting^4^. Because of such unique actions, DAH-IGRs are effective against insect pests that have already developed high resistance to conventional insecticides targeting the nervous system, such as organophosphates, carbamates, neonicotinoids, organochlorines, and pyrethroids.

Resistance to DAH-IGRs has already been reported in field populations of several lepidopteran pest species, such as obliquebanded leafroller, *Choristoneura rosaceana*, apple pandemic, *Pandemis pyrusana*^5,6^, and codling moth *Cydia pomonella*^7^. In addition, DAH-IGRs-resistant strains have been established by laboratory- or greenhouse-selection for beet armyworm *Spodoptera exigua*^8–10^ and diamondback moth, *Plutella xylostella*^11^. In some cases, detoxification enzymes such as cytochrome P450 monoxygenases (CYPs), have been suggested to be involved in the resistance to DAH-IGRs^12,13^. However, to the best of our knowledge, no modification of the target molecule of DAH-IGRs, i.e., EcR, has been reported as a factor for DAH-IGR resistance.

The smaller tea tortrix, *Adoxophyes honmai* is one of the most destructive insect pests in the tea fields of Japan. It developed strong resistance to DAH-IGRs, including tebufenozide, chromafenozide, and methoxyfenozide, in the early 2000s in Shizuoka prefecture, the largest tea production area in Japan^14^. Genetic studies revealed that tebufenozide-resistance is inherited as an autosomal and incompletely dominant trait controlled by polygenic factors^15^. However, the molecular and biochemical mechanisms of tebufenozide-resistance in *A. honmai* remain largely unknown.

As it is important to understand the mechanisms underlying insecticide resistance to develop better management systems for insect pests, the present study employed next-generation sequencing (NGS) techniques to identify the genes responsible for tebufenozide resistance in the smaller tea tortrix, *A. honmai*.

## Results

### Whole-genome sequencing of *A. honmai* and *de novo* genome assembly

A draft genome sequence of *A. honmai* was constructed using the following NGS data on the Kanaya1960-S strain (Supplementary Table S1). Raw sequence data with 45.8 Gb, 36.0 Gb, 36.4 Gb, and 39.7 Gb were obtained from paired-end libraries with insert sizes of 180 bp and 300 bp, and mate-pair libraries with insert sizes of 3 kb and 8 kb, respectively. *De novo* assembly of these data using the Platanus assmbler^16^ resulted in a draft genome assembly for *A. honmai* containing 469.62 Mb with N50 size of 188.71 kb (Supplementary Table S2). The number of scaffolds is 72,695 with average length of 6,460 bp, and 35.26 Mb of gaps (7.5% of the total assembly) were contained in the scaffolds. Assessment of the draft genome assembly by BUSCO2^17^ using the insecta_odb9 dataset showed that it covers 94.2% of complete BUSCO genes and 3.3% of fragmented BUSCO genes, whereas only 2.5% of BUSCO genes were missing (Supplementary Table S3), indicating a good quality of the draft genome assembly.

### Construction of a reference transcriptome assembly for *A. honmai*

A reference transcriptome assembly for *A. honmai* was constructed from pooled RNA sequencing (RNA-seq) data of a susceptible strain (Kanaya1960-S) and a resistant strain (Yui2012-R) (Supplementary Table S1). Basic statistics of this assembly are shown in Supplementary Table S4. In total, 105,001 contigs (Trinity transcripts) were obtained in this assembly and used as reference transcripts throughout the study. These contigs were further clustered into 63,454 genes (Trinity genes). Trinity genes were used for calculating gene expression levels for differentially expressed genes (DEGs) analysis.

### Double digest restriction-site associated DNA analysis and construction of a linkage map

The loci responsible for tebufenozide resistance were mapped onto the genome using double digest restriction-site associated DNA (ddRAD-seq) analysis^18^. After cleaning the 555.2 million raw reads (56.0 Gb) obtained from a parental female (Yui2012-R), a parental male (Kanaya1960-S), and 92 F_2_ individuals that survived the tebufenozide treatment, 526.7 million clean reads were assigned to each individual and used for further analyses. In total, 20,313 single nucleotide polymorphism (SNP) markers were identified between the parental female and male using Stacks modules^19^, and 945 SNP markers were genotyped on 85 or more F_2_ survivors (Supplementary Excel File S1).

Using JoinMap^20^, a linkage map containing 30 linkage groups (LGs) with a total size of 1859.2 cM was generated using 927 of the 945 SNP markers (Supplementary Fig. S1 and Excel File S1). These 927 SNP markers were mapped on 579 unique positions in the linkage map. Although the chromosome number of *A. honmai* has not yet been determined, the number of LGs was consistent with that of an allied species, *Adoxophyes orana* (n = 30)^21^_._ Furthermore, corresponding chromosomes in *Bombyx mori* were identified for all the LGs by mapping the SNP markers on *B. mori* chromosomes using the basic local alignment search tool (BLAST) (Supplementary Table S5). Of these, two corresponding *A. honmai* LGs were identified for three *B. mori* chromosomes, 11 (LG19 and LG23), 23 (LG6 and LG28), and 24 (LG13 and LG14), which is consistent with previous reports that these three chromosomes were generated by ancestral chromosomal fusion events of lepidopteran species with n = 31 karyotype^22–24^. On the other hand, two corresponding *B. mori* chromosomes, 1 (Z) and 15, were identified for *A. honmai* LG1 (Supplementary Table S5). The first half region of the LG1 corresponds with *B. mori* chromosome 15 and the second half region corresponds with *B. mori* chromosome 1 (Z), which is consistent with a Z-autosome fusion identified in another allied species, *Cydia pomonella*^25^. One-to-one correspondence was identified between remaining *A. honmai* LGs and *B. mori* chromosomes. These results verified that the linkage map of *A. honmai* was successfully established.

### Identification of candidate loci for tebufenozide-resistance by ddRAD-seq

**Figure 1:**
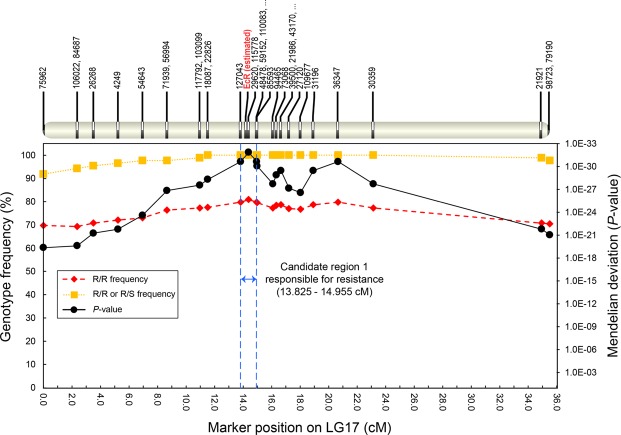
Candidate genomic region responsible for tebufenozide resistance on linkage group 17 (LG17) of *Adoxyophyes honmai*. Genotype frequency indicates the rate of F_2_ survivors after tebufenozide treatment with the same genotype to homozygous resistant strain (R/R; diamonds) or with the genotype of homozygous resistant strain or heterozygous strain (R/R or R/S; Squares) in each marker. The ID and position of SNP markers on LG17 are shown at the top. The *p*-value (circles) indicates the significance of deviation from Mendelian inheritance. The region between the two blue-dotted lines, encompassing the SNP marker 29620, indicates the candidate region responsible for tebufenozide resistance. *EcR* (bold red font) was estimated to be localized in this region by synteny analysis. See further details in Fig. 3.

**Figure 2:**
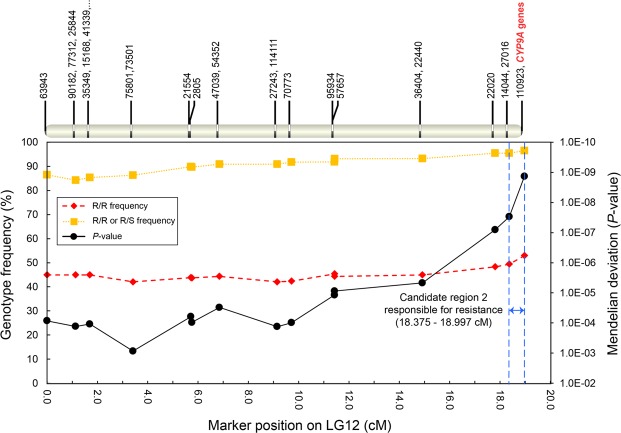
Candidate genomic region responsible for tebufenozide resistance on linkage group 12 (LG12) of *Adoxyophyes honmai*. Genotype frequency of R/R (diamonds) or “R/R or R/S” (squares), and *p*-value (circles) are the same as in Fig. 1. The ID and position of SNP markers on LG12 are shown at the top. The region between two blue-dotted lines, encompassing the most important marker 110923, indicates the second candidate region responsible for tebufenozide resistance. The most important marker 110923 was mapped on a *CYP9A* gene cluster (bold red font). See further details in Fig. 5.

Using this linkage map, we searched for the region responsible for tebufenozide-resistance. Because only F_2_ progenies that survived to high-dose tebufenozide exposure were used for this analysis, these survivors were expected to be either resistant homozygotes (R/R) or heterozygotes (R/S) for tebufenozide-resistance genes. Therefore, we expected genotypes of SNP markers tightly linked to resistant loci in the F_2_ progenies (i.e., resistant-type SNP markers) were either identical to that of the resistant mother (R/R) or heterogenic to that of the parents (R/S). To identify regions where resistant-type SNP markers were significantly enriched, we performed a chi-squared test for the 579 unique SNP markers mapped on the constructed linkage map. We identified SNP markers on 20 LGs with significant deviation from the Mendelian expected ratio (1:2:1) in F_2_ survivors (Supplementary Table S6). Of these, the SNP markers 29620 in LG17 and 110923 in LG12 showed significantly high genotype frequency of “R/R or R/S”: 100% and 96.7%, respectively. In particular, the genotype frequency of R/R in 29620 was 80.9%, which is much higher than that of marker 110923 (52.94%). We selected the genomic regions encompassing the most significant marker and its neighboring markers in the two LGs as candidate responsible regions for tebufenozide-resistance: 13.83 to 14.96 cM region on LG17 (neighboring markers are 127043 and 48478; Fig. 1) and 18.375 to 18.997 cM region on LG12 (neighboring marker is 14044; Fig. 2).

### Synteny analysis on LG17

**Figure 3:**
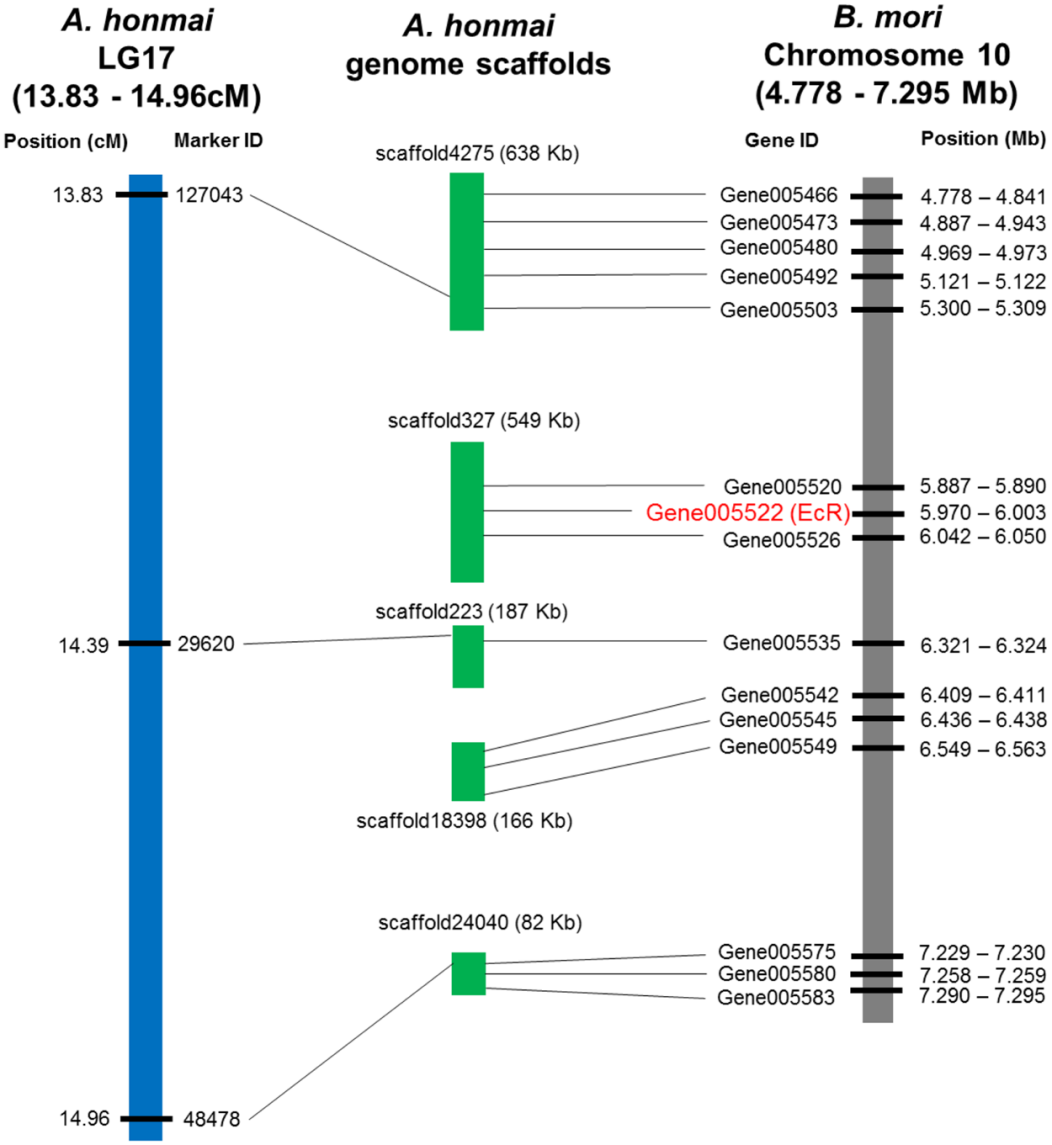
Conserved synteny between the LG 17 region of *Adoxophyes honmai* and the chromosome 10 of *Bombyx mori*. Three SNP markers on the candidate region responsible for tebufenozide resistance in LG17 (blue bar) were located on the three scaffolds (scaffold4275, scaffold223, and scaffold24040) of *A. honmai* genome (green bars). *B. mori* genes (shown on gray bar) mapped on the three scaffolds and other two scaffolds (scaffold327 and scaffold18398) revealed highly conserved synteny between the LG17 region and the 4.778–7.925 Mb region of *Bombyx mori* chromosome 10. Conserved synteny between scaffold327 and *B. mori* genes including EcR gene indicates that EcR gene of *A. honmai* is located on the region between the SNP marker 127043 and 29620 on LG17 (13.83–14.39 cM).

To obtain further information on the location of the loci responsible for tebufenozide resistance, we carried out synteny analysis on LG17. *A. honmai* and *B. mori* genomes revealed highly conserved synteny between the candidate genomic region on the LG17 of *A. honmai* (13.83 to 14.96 cM) and the 4.778 to 7.295 Mb region on chromosome 10 of *B. mori* (Fig. 3). Notably, *EcR*, which encodes a target molecule of tebufenozide^26^, is located on the middle of this syntenic region in *B. mori*, suggesting that the *EcR* of *A. honmai* (*AhEcR*) is also located on the corresponding genomic region on LG17 (13.83 to 14.39 cM) and is likely close to marker 29620 (Fig. 3).

### Comparison of ***EcR*** cDNA sequences between resistant and susceptible strains

Because the candidate responsible region in LG17 seems to contain *EcR*, we compared *EcR* cDNA sequences between susceptible and resistant strains based on RNA-seq data. The reference transcriptome assembly mentioned in section “Construction of a reference transcriptome assembly for *A. honmai*” was used for identifying the EcR cDNA sequence of *A. honmai* by tblastn search (https://blast.ncbi.nlm.hih.gov/) using the EcR peptide sequence of *B. mori* as query. A full-length cDNA sequence encoding the *EcR B1* isoform in *A. honmai* (*AhEcRB1*) was identified. Mapping the clean reads of individual RNA-seq data obtained from two resistant and two susceptible strains to the *AhEcRB1* sequence allowed identifying SNPs causing three non-synonymous amino acid substitutions (D26N, A415V, and K546R) in the deduced EcR protein sequence (Table 1). Among them, only SNP C1244T corresponding to A415V is consistent with DAH-resistance phenotypes in the four *A. honmai* strains, suggesting that this substitution is the most significant.

**Table 1:** Comparison of SNP causing non-synonymous amino acid substitution in *AhEcRB1* transcripts in local *Adoxyophyes honmai* strains.

Strains	SNP	G76A (GAT/AAT)	C1244T (GCT/GTG)	A1637G (AAG/AGG)
	Amino acid substitution	D26N	A415V	K546R
Yui2014-R	1	AAT**	GTG**	AGG**
	2	GAT/AAT*	GTG**	AGG**
	3	GAT/AAT*	GTG**	AGG**
Met-Sel-R	1	AAT**	GTG**	AGG**
	2	AAT**	GTG**	AGG**
	3	AAT**	GTG**	AGG**
Haruno2014-S	1	GAT	GCT	AAG
	2	GAT/AAT*	GCT	AAG/AGG*
	3	GAT/AAT*	GCT	AAG
Kanaya1960-S	1	GAT	GCT	AAG
	2	GAT	GCT	AAG
	3	GAT	GCT	AAG

Asterisks indicate the frequencies of Kanaya1960-S type SNPs (left side) in the cleaned RNA-seq reads as follows: ≥90% (no asterisk), 90% > ≥ 10% (*), and 10% > (**). Three biologically independent RNA-seq data were analyzed for each strain.

To validate the linkage of C1244T substitution in *AhEcR* with DAH resistance, the distribution of C1244T genotypes was compared with that of the SNP markers 29620 and 115778, which were tightly linked to tebufenozide-resistance, using the ddRAD-seq samples (Supplementary Fig. S2). Accordingly, the genotypes associated with these three SNP markers were exclusively correspondent in 89 F_2_ individuals with only one exception (98.8%), further supporting the idea that the SNP C1244T, which engenders A415V substitution in AhEcR protein (Supplementary Fig. S3), is critical for tebufenozide-resistance.

### Functional characterization of the A415 mutation in AhEcR

**Figure 4:**
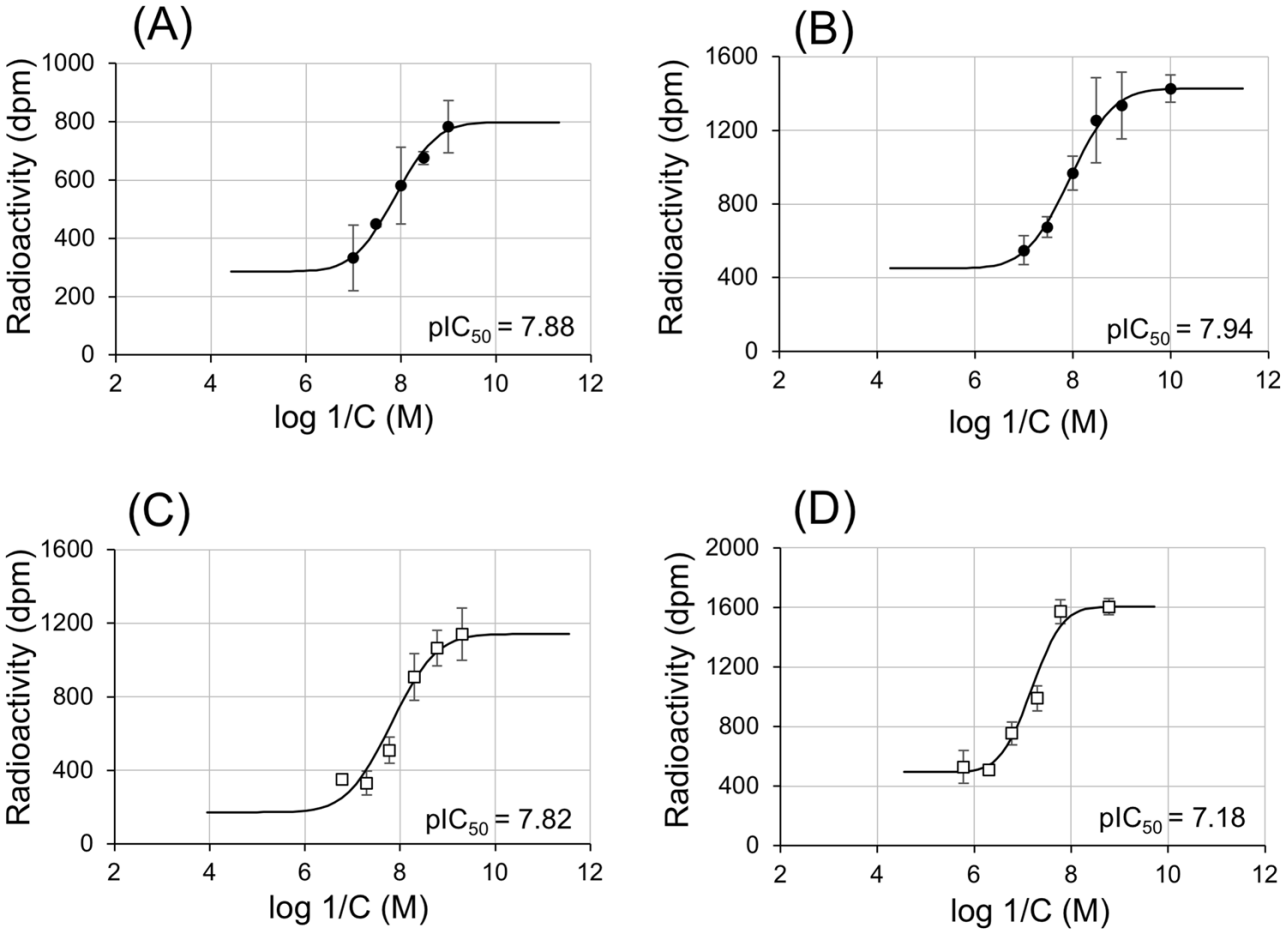
Concentration-response curves for ponasterone A (PonA) and tebufenozide treatments against EcRs in *Adoxophyes honmai*. Radioactivity obtained for the treatments with excess of PonA and carrier were set at 0% and 100%, respectively. From these response curves, concentrations that inhibit the binding of [^3^H]PonA to 50% (IC_50_, M) were determined by probit analysis for PonA and tebufenozide against EcRs of both wild and resistant strains. The binding activity of AhEcRB1 in tebufenozide-susceptible (**A**,**C**) or tebufenozide–resistant (**B**,**D**) strains to [^3^H]PonA was examined under exposure to cold PonA (**A**,**B**) or tebufenozide (**C**,**D**). Values are mean ± standard deviation (n = 3).

To verify the biochemical significance of AhEcR A415V substitution on tebufenozide-resistance, we cloned two *AhEcRB1* cDNAs, namely *AhEcRB1*_S and *AhEcRB1*_R, from the susceptible strain Kanaya1960-S and the resistant strain Yui2014-R, respectively. The nucleotide sequences of the two cDNAs showed three SNPs, namely T1149C and C1244T, and A1637G; however, because T1149C caused a synonymous substitution, A415V (C1244T) and K546R (A1637G) were the only difference at the protein level. The competition assays using *in vitro* translated proteins from the AhEcRB1/AhUSP1 heterodimeric complex and [^3^H]ponasterone A (PonA) revealed that this ecdysteroid, was equally competitive in AhEcRB1_S (pIC_50_ = 7.88; IC_50_ = 13.1 nM) and AhEcRB1_R (pIC_50_ = 7.94; IC_50_ = 11.5 nM) (Fig. 4A,B). In contrast, tebufenozide was about five-fold less competitive in AhEcRB1_R (pIC_50_ = 7.18; IC_50_ = 66.1 nM) than in AhEcRB1_S (pIC_50_ = 7.82; IC_50_ = 15.1 nM) (Fig. 4C,D). Thus, the binding affinity of PonA was similar between wild and resistant EcR, but the binding affinity of tebufenozide against resistant species was significantly lower (1/4–1/5) than that against wild type. These results revealed that, in the resistant strain, the A415V substitution decreased the affinity to tebufenozide without influencing the affinity to a natural ecdysteroid.

### Evaluation of candidate responsible genes in LG12

**Figure 5:**
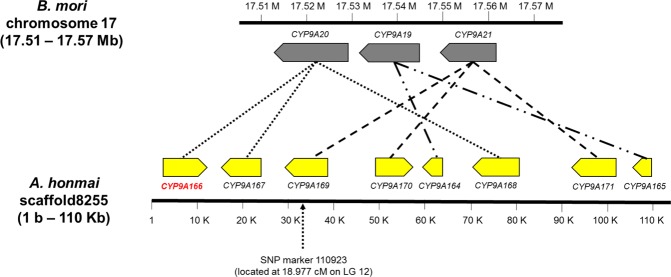
Conserved synteny between the LG12 region of *Adoxophyes honmai* and chromosome 17 of *Bombyx mori*. *Bombyx mori* chromosome 17 (17.51–17.57 Mb) contains three *CYP9A* genes. Scaffold 8255 of *A. honmai*, which corresponds to the candidate region responsible for tebufenozide resistance in LG12, contains eight *CYP9A* genes with high homology to the *B. mori CYP9A* genes. Orthologous genes between *B. mori* and *A. honmai CYP9A* genes are represented by dotted lines.

Next, we searched genes related to tebufenozide resistance in the second candidate responsible region in LG12. Genes of *A. honmai* on 110 kb genomic region (scaffold8255; 1 b–110 kb) including the significant SNP marker 110923, located at 18.977 cM in LG12, showed apparent conserved synteny with genes of *B. mori* on 60 kb genomic region (17.51–17.57 Mb) in chromosome 17 (chr17) (Fig. 5). Three P450 genes, namely *CYP9A20*, *CYP9A19*, and *CYP9A21*, were located on the 60 kb genomic region in chr17 of *B. mori*, while eight homologous *CYP9A* genes (Supplementary Table S7) were located in the corresponding genomic region of *A. honmai*. Notably, the SNP marker 110923 was located on an exon of gene *CYP9A1*6*9* (Fig. 5). These results suggested that one or more *CYP9A* homologs encoded in scaffold8255 might be relevant to the tebufenozide resistance in the LG12 region.

### Analysis of DEGs using RNA-seq data

**Figure 6:**
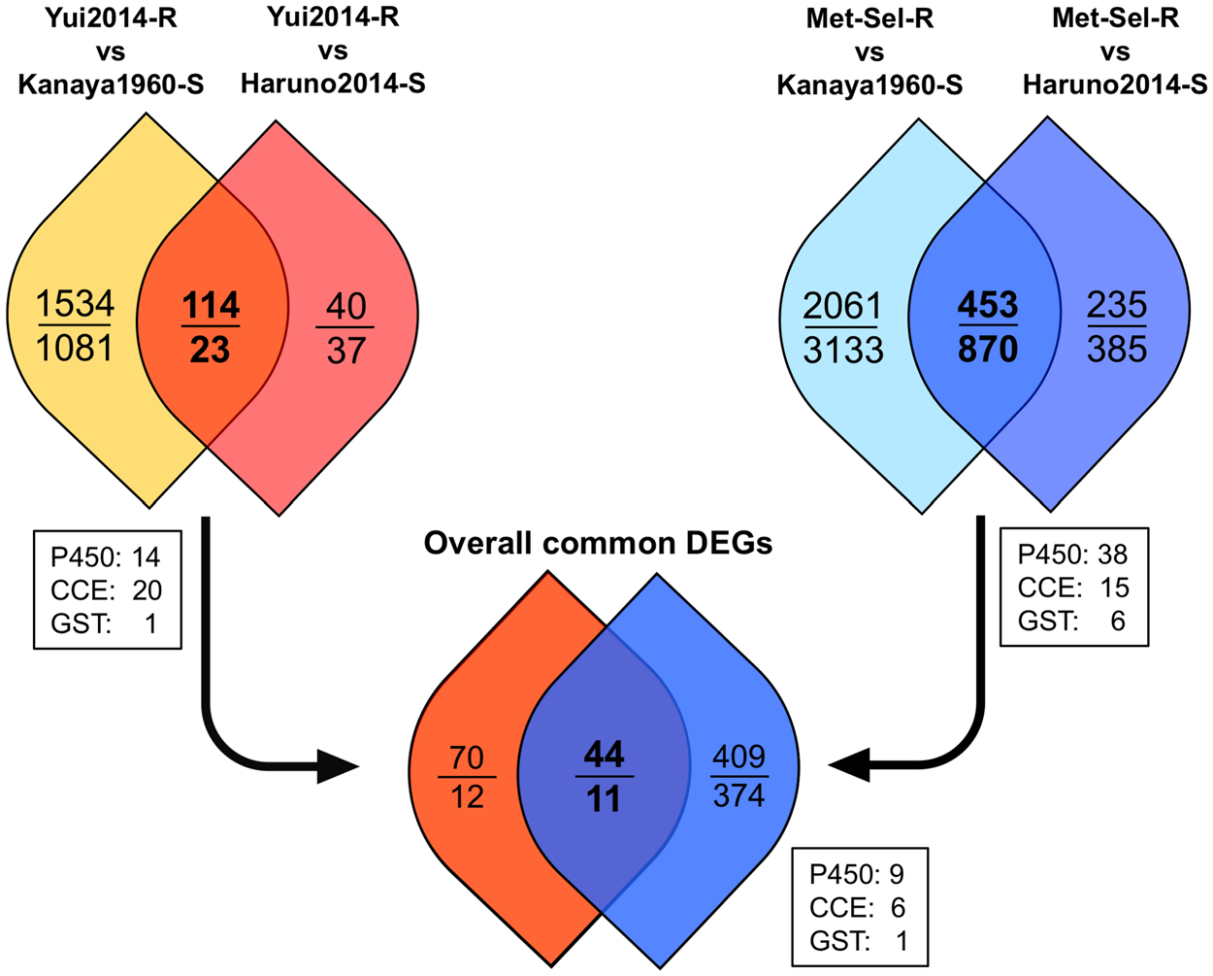
Schematic identification of differentially expressed genes (DEGs) between tebufenozide-resistant and -susceptible strains. Numbers within the circles indicate the up-regulated/down-regulated DEGs in resistant strains. Numbers within the boxes indicate common up-regulated metabolic enzyme genes in each comparison. Detailed information on common up- and down-regulated genes in each comparison are described in Supplementary Excel Files S2 and S3, respectively.

Generally, the increase of detoxification activity is one of the main causes for insecticide resistance. To identify tebufenozide-metabolyzing enzyme genes that are supposed to be commonly and highly expressed in resistant strains, we performed DEGs analysis. According to the strategy shown in Fig. 6, we compared the gene expression level calculated from RNA-seq data on two tebufenozide resistant strains (Yui2014-R and Met-Sel-R) and two susceptible strains (Kanaya1960-S and Haruno2014-S). First, we compared Yui2014-R to Kanaya1960-S and to Haruno2014-S, and identified 1,648/1,104 and 154/60 up-/down-regulated DEGs, respectively (Supplementary Fig. S4). By comparing these DEGs, 114 and 23 common up- and down-regulated DEGs were extracted, respectively (Fig. 6). Similarly, we compared Met-sel-R (LC_50_ to tebufenozide = 1,600 ppm) to Kanaya1960-S and to Haruno2014-S, and identified 2,514/4,003 and 688/1,255 up-/down-regulated DEGs, respectively (Supplementary Fig. S5). Of these, 453 and 870 common up- and down-regulated DEGs were in common between the two comparisons (Fig. 6). Finally, we compared the common DEGs obtained from the two comparisons and identified 44 and 11 overall common up- and down-regulated DEGs, respectively (Fig. 6 and Supplementary Excel File S2 and S3). The overall common up-regulated DEGs contained nine P450s, six choline/carboxylesterases (*AhCCE1-6*), and one glutathione *S*-transferase (*AhGST1*). No other P450, CCE, or GST genes were found in the final common down-regulated DEGs. Notably, *CYP9A169* and *CYP9A171*, likely localized near the tebufenozide-responsible region in LG12 (Fig. 5, Supplementary Table S7), were found in the overall common up-regulated DEGs. In contrast, no homologous coding genes of *B. mori* in chromosome 10 were found among these DEGs. These results further support that *CYP9A* homologs encoded in the candidate responsible region in LG12 might contribute to the tebufenozide resistance.

### Reverse transcription quantitative real-time PCR (RT-qPCR) analysis of *CYP9A* homologs

**Figure 7:**
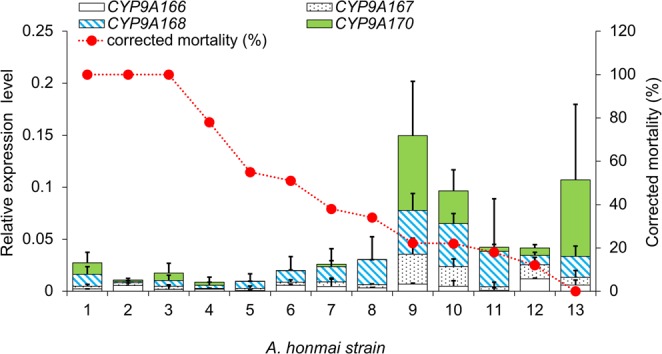
Relationship between the expression levels of four *CYP9A* genes and corrected mortality in 13 *Adoxophyes honmai* strains. Bars indicates the expression levels of four *CYP9A* genes relative to a reference gene (*rp49*) as obtained by quantitative real-time PCR (mean + standard deviation, n = 3). The relative expression levels of *CYP9A166* (open bars), *CYP9A167* (dotted bars), *CYP9A170* (filled bars), and *CYP9A168* (diagonal-striped bars) are indicated for each strain, along with corrected mortality (red dots). The numbers at the bottom indicate the strains of *A. honmia* listed in Supplementary Table S8.

The expression level of four *CYP9A* homologs, *CYP9A166*, *CYP9A167*, *CYP9A168*, and *CYP9A170* (Supplementary Table S7) was evaluated by RT-qPCR in 13 regional strains with different magnitude of tebufenozide-resistance (Supplementary Table S8). Each strain showed unique expression profiles in the four *CYP9A* genes. However, in general, the expression levels of *CYP9A* genes tended to be higher in the strains with lower corrected mortalities to tebufenozide (Fig. 7). Significant correlations were observed between the corrected mortality and the mRNA expression levels in *CYP9A167* and *CYP9A168* (*r* = −0.768 and −0.663, *p* < 0.05; Spearman correlation) (Supplementary Table S9). Furthermore, a high correlation was observed in the sum of four *CYP9A* genes (*r* = −0.7845, *p* = 0.0015). These results suggested that high expression of *CYP9A* family genes in the LG12 region is involved in the tebufenozide-resistance in *A. honmai*.

### RT-qPCR analysis of *CCEs* and *GST* homologs

We also examined the expression level of *AhCCE1*-*6* and *AhGST1* by RT-qPCR. The expression level of *AhCCE*s tended to be higher in resistant strains (Supplementary Fig. S6). In particular, the expression levels of *AhCCE2*, *AhCCE3*, *AhCCE4*, and the sum of six *AhCCE* genes showed significant correlations with the corrected mortality (Supplementary Table S10). By contrast, no significant correlation was found between the expression level of the *AhGST1* and the corrected mortality (Supplementary Fig. S7, Table S10). These results suggested that higher expression of several *CCE* genes are involved in the tebufenozide resistance in *A. honmai*, wherease the involvement of *GST* was unclear.

## Discussion

With the aid of a series of NGS analyses, we have conducted genome-wide searching of the genes responsible for tebufenozide resistance in *A. honmai* and identified candidate genes that implicate two resistant mechanisms. One is the decreased sensitivity to tebufenozide caused by an amino acid substitution in the target molecule EcR, and the other is increased detoxification activity caused by the overexpression of multiple *CYP9A* and *CCE* genes.

First, ddRAD-seq analysis identified a candidate locus in LG17. The following synteny analysis between *A. honmai* and *B. mori* using the newly-generated draft genome sequences and linkage map of *A. honmai* revealed that the LG17 corresponds to chromosome 10 of *B. mori* and the locus contained the *EcR* gene. Successively, the RNA-seq analysis identified the non-synonymous SNP causing the A415V amino acid substitution in the ligand-binding domain of the EcR protein, and direct cDNA sequencing analysis clarified its tight linkage with tebufenozide resistance. Finally, biochemical analysis using *in vitro*-translated EcR protein suggested that the affinity to tebufenozide was significantly decreased in the EcR of resistant strains, whereas the affinity to the endogenous ligand, 20E, was not. Collectively, we conclude that the EcR A415V substitution is the major factor accounting for the expression of tebufenozide resistance in *A. honmai*.

This A415V substitution may reasonably explain the decline of susceptibility to DAH-IGRs without changing the vital function of EcR. The crystal structure of EcR in tobacco budworm, *Heliothis virescens*, revealed that the amino acid residues critical for binding to a natural, plant-derived ecdysteroid (PonA) are widely conserved among insects^26^. In contrast, different amino acid residues were used for binding to a lepidopteran specific DAH (BYI06830) with similar structure to tebufenozide^27^. The A415 residue in *A. honmai* EcR (equivalent to A386 residue in *H. virescens* EcR), neighbours R413, which directly interacts with ecdysteroid in the ligand binding pocket, and V414, which is only conserved among lepidopteran EcRs^28^ (Supplementary Fig. S3). It is thus plausible that A415V substitution modifies the local structure of the ligand binding pocket to selectively alleviate the interaction with DAH-IGRs but not with natural ligands. Further studies on molecular dynamics and crystal structure analysis are required to elucidate the interaction between A415V residues and DAH-IGRs.

The ddRAD-seq analysis identified the second candidate locus in LG12 and the synteny analysis revealed that it corresponds to the region in *B. mori* chr 17 containing three *CYP9A* genes. This LG12 region contains at least eight *CYP9A* genes including two *CYP9A19*-like (*CYP9A164* and *CYP9A165*), three *CYP9A20*-like (*CYP9A166*, *CYP9A167*, and *CYP9A168*), and three *CYP9A21*-like (*CYP9A169*, *CYP9A170*, and *CYP9A171*) genes, suggesting the occurrence of gene duplication in the three *CYP9A* genes (Fig. 5). Similar extensive duplication events in the *CYP9A* gene cluster were reported in the three noctuid moths, *Spodoptera litura*^29^, *Spodoptera frugiperda*, and *Helicoverpa armigera*^30^, which have developed high resistance to many insecticides.

The DEG and RT-qPCR analyses further confirmed the involvement of these *CYP9A* genes in tebufenozide resistance. In general, enzymatic detoxification is one of the major mechanisms for insecticide resistance and P450s play the most important roles. Increased levels of P450 gene expression in resistant insect strains have been reported in numerous cases^31^. In this study, DEG analyses showed that *CYP9A166* was commonly upregulated in the resistant strains. Furthermore, RT-qPCR analysis revealed that the expression levels of two *CYP9A* genes (*CYP9A167* and *CYP9A168*), and sum of four *CYP9A* family genes encoded in the LG12 region were significantly correlated with the magnitude of resistance (Supplementary Table S9). Altogether, the tebufenozide resistance in *A. honmai* is, at least in part, attributable to the elevated detoxification activity of the enzymes abundantly produced from the highly expressed *CYP9A* genes.

In addition to *CYP9A* genes, the DEG and RT-qPCR analyses indicated that higher expression of *CCE* genes are also involved in the tebufenozide resistance. The relative importance of each *CYP9A* and *CCE* gene on tebufenozide resistance remains unclear. We must note that some field populations of *A. honmai* in Shizuoka prefecture had acquired strong resistance to various insecticides, such as carbamates, organophosphates, and pyrethroids^14^. In addition, development against diamide insecticides, including flubendiamide and chlorantraniliprole, have been recently reported^32^. The Yui2014-R strain used for the DEG analysis performed in the present study was also resistant to both DAH-IGRs and diamide insecticides (Uchiyama *et al*., unpublished). P450s and CCEs are known to play a substantial role in the degradation of a number of insecticides, such as carbamates, organophosphates, pyrethroids, and diamides^33–36^. Although we compared two tebufenozide-resistant strains with two tebufenozide-susceptible strains having different genetic backgrounds to reduce the possibility to pick-up genes unrelated to tebufenozide resistance, it is not completely deniable that some of the highly expressed P450s and CCEs were related to resistance to other insecticides. For further determining the role of each *CYP9A* and CCE gene in tebufenozide resistance, substrate specificity and catalytic activity of individual enzymes will need to be analysed using recombinant proteins.

The results obtained in the present study are consistent with the mode of inheritance of tebufenozide resistance reported in a previous study: autosomal, incompletely dominant, and controlled by polygenic factors^15^. Both *EcR* and *CYP9A*s were found to be located in autosomes. It is likely that the decline of binding activity in EcR by the amino acid substitution is a recessive trait, while the increase of detoxification activities by the overexpression of CYP9A and CCE enzymes is a dominant trait. Thus overlapping of these two traits likely appears incomplete dominance.

We believe that the modification of EcR is the main factor while *CYP9A* overexpression is a supplemental factor, which enhances the former action, based on the following reasons. The correlations between the *CYP9A* and *CCE* gene expression levels and the magnitude of tebufenozide-resistance were low to moderate (Supplementary Table S9 and S10). In addition, the expression levels of *CYP9A* and *CCE* genes in some resistant strains (e.g. strains 6, 7, 8, 11, and 12 for *CYP9A*s; strains 4, 5, and 8 for *CCE*s) were as low as in a susceptible strain (Strain 1–3) (Fig. 7), suggesting that higher expression of *CYP9A* and *CCE* genes is dispensable in some resistant strains. In contrast, our preliminary analysis revealed that the frequency of A415V substitution was very highly correlated with the magnitude of tebufenozide-resistance in these populations (*r* = −0.9049, *P* = 0.0003, n = 11, Spearman correlation coefficient). Further confirmation of the importance of the EcR substitution in tebufenozide-resistance using more local strains will be reported elsewhere (Uchibori-Asano *et al*., in preparation).

In conclusion, using NGS techniques, we clarified molecular mechanisms of tebufenozide resistance in *A. honmai*. The combination of ddRAD-seq, RNA-seq, and genome sequence analyses was highly efficient to reveal tebufenozide resistance mechanisms. The discovery that A415V substitution in EcR causes tebufenozide resistance will be valuable for developing management systems not only for *A. honmai* but also for other lepidopteran pests.

## Methods

### Insects

Smaller tea tortrix, *A. honmai*, were collected from tea fields in different regions in Japan, and maintained in the laboratory on an artificial diet (Insecta LFS, NOSAN) at 25 °C under 16L8D conditions^14^. The strains used for genome sequencing, ddRAD-seq, and RNA-seq analyses are listed in Supplementary Table S1. Strain Kanaya1960-S was the representative tebufenozide-susceptible strain, and it was collected in the 1960s and maintained in the laboratory since then without exposure to any insecticides. The Met-sel-R was established in the laboratory by selection on an artificial diet dipped into methoxyfenozide for 22 generations, as indicated in Supplementary Table S11. Strains used for RT-qPCR of cytochrome P450 genes are listed in Supplementary Table S8. Bioassays for determining corrected mortality of the strains are described in Supplementary Methods.

### Whole genome sequencing and *de novo* genome assembly

Genomic DNA was extracted from 24 adult males of the Kanaya1960-S strain using the DNeasy Blood and Tissue kit (QIAGEN). Because the W chromosome of Lepidoptera usually contains highly repetitive sequences^37^, we avoided extracting DNA from females (W/Z), and only used males (Z/Z) to increase assemble quality. Moreover, two-rounds of inbreeding, starting from a single pair, were carried out to improve genomic DNA homogeneity. Two paired-end (insert size is 180 bp and 500 bp) and two mate-pair (insert size is 3 kb and 8 kb) libraries were generated based on the manufacturer’s protocol (https://www.illumina.com/). Each library was sequenced on one lane of the Illumina HiSeq2000, generating 101-bp paired-end and mate-pair reads, respectively (Macrogen Japan Corp.). The paired-end reads and mate-pair reads were cleaned by platanus_trim version 1.0.7 and platanus_internal_trim version 1.0.7 (http://platanus.bio.titech.ac.jp/pltanus_trim), respectively. Platanus version 1.2.4^16^ was used for generating a draft genome assembly of *A. honmai*. First, all the cleaned paired-reads were merged and *de novo* assembled using the assemble command in Platanus. Second, scaffolding of all the cleaned paired-end and mate-pair reads was performed on the assembled contigs using the scaffold command in Platanus. Finally, gap-closing using all the cleaned reads was performed on the scaffolds using the gap_close command in Platanus, and scaffolds with lengths of 200 bp or more were extracted. The completeness of the generated genome assembly was assessed by BUSCO2^17^ using insecta_odb9 (1658 BUSCO genes).

### Transcriptome assembly by RNA-seq

Second-instar larvae of Yui2012-R and Kanaya1960-S strains were fed with tea leaves dipped in tebufenozide solution (LC_10_ = 46.9 ppm for Yui2012-R and 2.67 ppm for Kanaya1960-S) for 24 h, and surviving larvae were collected. Total RNA was extracted from the larvae using the RNeasy Plus Mini kit (QIAGEN). Two larvae were used in each extraction, which was performed in triplicate. A cDNA library was constructed for each Total RNA sample and then sequenced on the Illumina HiSeq2000 platform by Hokkaido System Science Co. Ltd using the 101-bp paired-end mode. The obtained reads were cleaned using Trimmomatic 0.32^38^. All the clean reads were merged and *de novo* assembled by Trinity r20140717^39^. Each generated RNA-seq contig was compared with the National Center for Biotechnology Information non-redundant (NCBI-nr) protein database by blastx search (e-value < 1e-3) and top-hit information was extracted. The coding sequence (CDS) was predicted for each RNA-seq contig by TransDecoder bundled with the Trinity r20140717. The predicted CDSs were compared with Pfam domain database by HMMER3^40^ and homologous conserved domains were identified. The annotated RNA-seq contigs were used as a reference transcriptome assembly for DEG analysis and linkage analyses. The details of “Analysis of DEGs based on RNA-seq” are described in Supplementary Methods.

### ddRAD-seq analysis and construction of linkage groups (LGs)

A resistant strain (Yui2012-R) female and a susceptible strain (Kanaya1960-S) male were crossed, and the resulting F_1_ siblings were randomly crossed to obtain F_2_ individuals. Second-instar F_2_ larvae were fed tea leaves dipped in tebufenozide solution (150 ppm) and surviving larvae (92 individuals) were collected after 10 days (fifth-instar larvae). Genomic DNA was extracted individually from the female and male parents and from the 92 F_2_ larvae using the DNA blood and tissue kit (QIAGEN). The ddRAD-seq library was constructed using all genomic DNAs according to the method described in Peterson *et al*.^18^. Two restriction enzymes, EcoRI and MspI, were used for digesting genomic DNAs. To recognize the genomic DNA of each individual, a unique pair of inline barcodes (ligated onto the EcoRI-associated DNA) and index barcodes (located within sequencing adapters) were assigned to each sample. The ddRAD-seq library was sequenced on two lanes of the Illumina HiSeq2000, generating 101-bp paired-end reads (Macrogen Japan Corp.). Sequence data were processed using Stacks 1.26 modules^19^. Reads obtained for each individual were first demultiplexed and cleaned using the process_radtags module. A catalog of SNP markers was then generated by processing the cleaned reads successively using ustacks (−m = 3 and −M = 3 options), cstacks (−n = 2 option), and sstacks. Finally, F_2_ individual genotypes were calculated in the genotypes module. The DNA markers generated were then filtered by in-house Perl scripts, which removed the markers in less than 85 F_2_ individuals and the F_2_ individuals genotyped with less than 80 percent of the remaining markers. The remaining SNP markers were used for linkage analysis in JoinMap 4.1^20^. LGs were clustered with a logarithm of odds score threshold = 8, and the DNA markers in each LG were ordered using a maximum likelihood mapping algorithm with default parameters. The details of the analyses using the ddRAD-seq data and the linkage map, namely “Identification of corresponding *B. mori* chromosomes for *A. honmai* linkage groups” and “Identification of candidate loci responsible for tebufenozide resistance by ddRAD-seq analysis”, and “Determination of AhEcR A415V genotypes in ddRAD samples”, are described in Supplementary Methods.

### Binding activity of tebufenozide and PonA to *in vitro* translated EcR/USP complex

Using our previously published methods^41,42^, AhEcR_S, AhEcR_R, and AhUSP-1 were translated *in vitro* from their corresponding cDNAs inserted in the plasmid pTnT using TNT Coupled Reticulocyte Lysate Systems (Promega Corp.), from their corresponding cDNAs inserted in the plasmid pTnT according to the vendor protocol. The details of “Cloning and sequencing of *AcEcRB1* cDNAs and construction of expression plasmids” are described in Supplementary methods. *In vitro* translated AhEcR (_S or _R) and AhUSP-1, test compound, and PonA (25,000 dpm/tube) were mixed in the siliconized tube. After incubating the mixture at 25 °C for 60 mins, the reaction mixture was immediately filtered through a glass filter (GF-75; ADVANTEC) with the aid of a vacuum pump. Filters were washed three times with washing buffer and transferred to a vial containing 3 ml of Aquasol-2 (Perkin-Elmer) to measure the radioactivity in an Aloka LSC-6100 liquid scintillation counter (Aloka). A 1000-fold excess of unlabeled PonA was added to measure nonspecific binding, and total binding was obtained for the treatment with a carrier (DMSO or EtOH).

### RT-qPCR

The transcript levels of four *CYP9A*s (*CYP9A166*, *CYP9A167*, *CYP9A168*, and *CYP9A170*), six *CCE*s (*AhCCE1-AhCCE6*), and a *GST* (*AhGST1*) in the larvae of 13 *A*. *honmai* regional strains listed in Supplementary Table S8 were quantified by RT-qPCR. The details for RT-qPCR analyses were described in “RNA extraction and cDNA preparation for RT-qPCR”, “RT-qPCR for *P450* genes”, and “RT-qPCR for *CCE* and *GST* genes” in Supplementary Methods.

The NGS raw sequence data and assembled sequence data used in this study were deposited in the DNA Data Bank of Japan (DDBJ) with accession numbers as follows: raw sequence data used for *A. honmai* genome assembly, DRA006990; raw sequence data used for *A. honmai* transcriptome assembly, DRA006991; raw sequence data used for DEG analyses, DRA006989 (Kanaya 1960-S, Haruno2014-S, and Met-Sel-R strains) and DRA007049 (Yui2014-R strain); raw sequence data used for ddRAD-seq analyses, DRA007074; scaffold sequences of the genome assembly, BHDV01000001-BHDV01072695 (72695 entries); contig sequences of the transcriptome assembly, IADH01000001-IADH01105001 (105001 entries), including *AhCCE1*, IADH01020002; *AhCCE2*, IADH01033190; *AhCCE3*, IADH01033194; *AhCCE4*, IADH01048962; *AhCCE5*, IADH01057165, *AhCCE6*, IADH01072630; and *AhGST1*, IADH01038181. The full open reading frame cDNA sequences were deposited in the DDBJ with accession numbers as follows: *AhEcRB1_R*, LC209228; *AhEcRB1_S*, LC209229; *AhUSP-1*, LC209230; *CYP9A164*, LC388681; *CYP9A165*, LC388682; *CYP9A166*, LC388683; *CYP9A167*, LC388684; *CYP9A168*, LC388685; *CYP9A169*, LC388686; *CYP9A170*, LC388687; *CYP9A171*, LC388688; and *rp49*, LC388758.

## Supplementary information

Supplementary Information

Supplementary Excel File S1

Supplementary Excel File S2

Supplementary Excel File S3
