# Supplementary material for: Genome-wide Identification of Tebufenozide Resistant Genes in the smaller tea tortrix, Adoxophyes honmai (Lepidoptera: Tortricidae)
Source: Sci Rep. 2019 Mar 12;9:4203. doi: 10.1038/s41598-019-40863-5 (PMC6414682; doi:10.1038/s41598-019-40863-5)
Supplement: Supplementary file 1 — Supplementary Information [file 41598_2019_40863_MOESM1_ESM.docx]

**Supplementary Information**

**This file contains Supplementary Table S1-S12, Supplementary Methods, Supplementary References, and Supplementary Figure S1-S7.**

**Genome-wide Identification of Tebufenozide Resistant Genes in the smaller tea tortrix, *Adoxophyes honmai* (Lepidoptera: Tortricidae)**

Miwa Uchibori-Asano^1^, Akiya Jouraku^1^, Toru Uchiyama^2^, Kakeru Yokoi^1^, Gaku Akiduki^3^, Yoshitaka Suetsugu^1^, Tetsuya Kobayashi^1^, Akihito Ozawa^2^, Saki Minami^4^, Chiharu Ishizuka^4^, Yoshiaki Nakagawa^4^, Takaaki Daimon^1,5^ ＆ Tetsuro Shinoda^1^

^1^Institute of Agrobiological Sciences, National Agriculture and Food Research Organization (NARO), Tsukuba, Ibaraki, 305-8634, Japan

^2^Tea Research Center, Shizuoka Research Institute of Agriculture and Forestry, Kurasawa, Kikugawa, Shizuoka, 439-0002, Japan

^3^Kyushu Okinawa Agricultural Research Center, National Agriculture and Food Research Organization (NARO), Kumamoto 861-1192, Japan

^4^Division of Applied Life Sciences, Graduate School of Agriculture, Kyoto University, Kitashirakawa Oiwakecho, Sakyo-ku, Kyoto 606-8502, Japan

^5^Department of Applied Biosciences, Graduate School of Agriculture, Kyoto University, Kitashirakawa Oiwakecho, Sakyo-ku, Kyoto 606-8502, Japan

Miwa Uchibori-Asano and Akiya Jouraku contributed equality to this work.

Correspondence and requests for materials should be addressed to T.S. (email: shinoda@affrc.go.jp)

**Table S1. *Adoxophyes* *honmai* strains used for genome sequencing, ddRAD-seq, and RNA-seq analyses.**

| **Name** | **Collection site** | **Prefecture** | **Latitude, Longitude** | **Collection year** | **LC_50_ to tebufenozide (ppm)** | **Analyses** |
| --- | --- | --- | --- | --- | --- | --- |
| Kanaya1960-S | Kanayashishidoi,  Shimada | Shizuoka | N34.80, E138.13 | 1960s | 4.46 | Genome sequencing  ddRAD-seq  RNA-seq (Transcriptome assembly)  RNA-seq (DEG analysis) |
| Haruno2014-S | Harunocho,  Hamamatsu | Shizuoka | N34.97, E137.95 | 2014 | 5.52 | RNA-seq (DEG analysis) |
| Yui2012-R | Yui, Shimada | Shizuoka | N34.81, E138.19 | 2012 | 1073 | ddRAD-seq  RNA-seq (Transcriptome assembly) |
| Yui2014-R | Yui, Shimada | Shizuoka | N34.81, E138.19 | 2014 | 359 | RNA-seq (DEG analysis) |
| Met-Sel-R (F_23_) | Kurasawa,  Kikugawa | Shizuoka | N34.78, E138.14 | 2010 | 1600  281* | RNA-seq (DEG analysis) |

*LC_50_ to methoxyfenozide.

**Table S2. Basic statistics of *Adoxophyes* *honmai* genome sequences.**

Number of scaffolds 72,695

Total length (total nucleotide numbers) 469,621,355bp

N50 188,712 bp

Number of gaps (N’s) 35,261,528 bp

Average length of scaffolds 6,460 bp

Maximum length of scaffolds 1,334,760 bp

*A. honmai* genome sequence were obtained by *de novo* assembly of Illumina HiSeq 2000 101 bp paired-end reads (insert size is 180 bp and 300 bp) and mate pair reads (insert size is 3 kb and 8 kb) using Platanus 1.2.4.

**Table S3. Result of assessment of *Adoxophyes* *honmai* draft genome assembly by BUSCO2.**

| Complete BUSCOs | 1561 (94.2%) |
| --- | --- |
| Complete and single-copy BUSCOs | 1543 (93.1%) |
| Complete and duplicated BUSCOs | 18 (1.1%) |
| Fragmented BUSCOs | 54 (3.3%) |
| Missing BUSCOs | 43 (2.5%) |

insecta_odb9 dataset including 1658 BUSCO genes were used. The numbers of covered BUSCO genes (complete or fragmented) and missing BUSCO genes by the draft genome assembly are shown. Complete BUSCOs are classified into single-copy BUSCOs (only one complete copy was found) and duplicated BUSCOs (two or more complete copies were found).

**Table S4. Basic statistics of *Adoxophyes* *honmai* reference transcriptome assembly.**

Statistics of all contigs (Trinity transcripts)

Number of contigs 105,001

Total bases (total nucleotide numbers) 130,442,924 bp

GC content percent 43.27 %

N50 2,534 bp

Average length of contigs 1,242 bp

Maximum length of contigs 35,339 bp

Minimum length of contigs 201 bp

Number of blastx (NCBI-nr) hits 61,407

Number of predicted ORFs 40,945

Average length of ORFs 1,447 bp

Number of HMMER3 hits 30,256

Statistics of all genes (Trinity genes)

Number of genes 63,454

Total bases 53,879,860 bp

GC content percent 42.06 %

N50 1,795 bp

Average length of genes 849 bp

Number of blastx (NCBI-nr) hits 20,205

Number of predicted ORFs 16,983

Average length of ORFs 1,299 bp

Number of HMMER3 hits 12,599

Reference contigs were obtained by de novo assembly using Trinity from merged RNA-seq data of Kanaya1960-S and Yui2012-R. Only longest transcript per Trinity gene was used for calculating the statistics of the genes.

**Table S5. Linkage groups (LGs) of *Adoxophyes homai* and corresponding chromosomes (chr.) of *Bombyx mori***

| *B. mori* chr. | *A. honmai* LG | Scaffolds hit to *B. mori* chr. | RNA-seq contigs hit to *B. mori* chr. |
| --- | --- | --- | --- |
| chr1 (Z) | LG1 (second half) | 19 | 2 |
| chr2 | LG27 | 1 | 0 |
| chr3 | LG24 | 9 | 2 |
| chr4 | LG15 | 17 | 6 |
| chr5 | LG5 | 27 | 10 |
| chr6 | LG2 | 14 | 1 |
| chr7 | LG11 | 7 | 2 |
| chr8 | LG29 | 10 | 6 |
| chr9 | LG8 | 15 | 4 |
| chr10 | LG17 | 17 | 4 |
| chr11 | LG19 | 1 | 2 |
| chr11 | LG23 | 13 | 3 |
| chr12 | LG25 | 12 | 6 |
| chr13 | LG9 | 21 | 4 |
| chr14 | LG3 | 8 | 2 |
| chr15 | LG1 (first half) | 21 | 6 |
| chr16 | LG7 | 17 | 4 |
| chr17 | LG12 | 3 | 2 |
| chr18 | LG4 | 10 | 3 |
| chr19 | LG20 | 14 | 6 |
| chr20 | LG26 | 6 | 3 |
| chr21 | LG21 | 10 | 6 |
| chr22 | LG22 | 15 | 2 |
| chr23 | LG6 | 13 | 6 |
| chr23 | LG28 | 2 | 1 |
| chr24 | LG13 | 4 | 1 |
| chr24 | LG14 | 1 | 1 |
| chr25 | LG16 | 12 | 3 |
| chr26 | LG10 | 6 | 0 |
| chr27 | LG18 | 3 | 0 |
| chr28 | LG30 | 1 | 2 |

*A. honmai* scaffolds and RNA-seq contigs including one or more RAD-loci were identified for each LG by blastn search. Corresponding *B. mori* chromosome was estimated for each *A. honmai* LG based on number of top hit *A. honmai* scaffolds and RNA-seq contigs. Three pairs of LGs, LG19 and LG23, LG6 and LG28, LG13 and LG14 correspond with *B. mori* chr11, chr23, and chr24, respectively. The first half of the LG1 corresponds to the *B. mori* chr15 whereas the second half of the LG1 corresponds to the *B. mori* chr1 (Z).

**Table S6. Linkage groups (LGs) and corresponding SNP markers showing significant deviation from Mendelian inheritance, according to chi-squared test results.**

| **LG** | **Number of unique SNP markers** | **Most significant SNP marker** | **Chi-squared test** | | **Genotype frequency** | |
| --- | --- | --- | --- | --- | --- | --- |
|  |  |  | **χ^2^ (1:2:1)** | **p-value** | **R/R (%)** | **R/R or R/S (%)** |
| LG17 | 22 | 29620 | 146.66 | 1.42E-32 | 80.9 | 100 |
| LG12 | 15 | 110923 | 40.85 | 1.35E-09 | 52.94 | 96.47 |
| LG7 | 23 | 51696 | 18.1 | 1.17E-04 | 45.35 | 84.88 |
| LG11 | 19 | 59384 | 21.45 | 2.20E-05 | 23.26 | 53.49 |
| LG18 | 4 | 20799 | 25.42 | 1.80E-06 | 21.18 | 50.59 |
| LG26 | 10 | 77895 | 26.45 | 3.02E-06 | 16.47 | 50.59 |
| LG8 | 20 | 110301 | 28.1 | 5.83E-07 | 20 | 49.41 |
| LG9 | 19 | 109607 | 28.71 | 7.91E-07 | 17.65 | 49.41 |
| LG29 | 14 | 25057 | 33.56 | 5.16E-08 | 15.29 | 47.06 |
| LG6 | 27 | 81158 | 39.52 | 2.62E-09 | 21.84 | 45.98 |
| LG16 | 22 | 128902 | 37.75 | 6.35E-09 | 20 | 45.88 |
| LG27 | 7 | 78356 | 38.38 | 4.63E-09 | 15.12 | 45.35 |
| LG24 | 21 | 44085 | 42.94 | 4.74E-10 | 19.77 | 44.19 |
| LG19 | 6 | 78595 | 47.02 | 6.16E-11 | 16.09 | 42.53 |
| LG14 | 8 | 11938 | 61.44 | 4.55E-14 | 25.88 | 40 |
| LG4 | 24 | 46792 | 66.65 | 3.37E-15 | 17.24 | 36.78 |
| LG20 | 27 | 116614 | 70.08 | 6.06E-16 | 16.47 | 35.29 |
| LG21 | 23 | 59891 | 73.23 | 1.25E-16 | 12.94 | 34.12 |
| LG3 | 16 | 49772 | 76.84 | 2.06E-17 | 16.28 | 33.72 |
| LG1 | 60 | 126889 | 82.75 | 1.07E-18 | 8.99 | 32.58 |

The significance of the deviation from Mendelian inheritance (1:2:1 genotypic proportion among F_2_ individuals) was calculated for each SNP marker using the chi-squared test (degrees of freedom = 2). Bonferroni corrected significance threshold (p < 0.05/N; N is the number of unique SNP markers on each LG) was used for identifying significantly deviated SNP markers. Genotype frequency of “R/R” is the proportion of F_2_ individuals that survived tebufenozide exposure with homozygous resistant strain genotype for each marker. Similarly, genotype frequency of “R/R or R/S” is the proportion of F_2_ individuals that survived tebufenozide exposure with homozygous resistant strain or heterozygous genotype for each marker. Results of the chi-squared test and genotype frequencies of the most significant SNP marker in each LG are shown. LGs were sorted in order of decreasing genotype frequency of “R/R or R/S”.

**Table S7. Cytochrome P450 genes on LG12 similar to *CYP9A* genes in *Bombyx mori.***

| **Gene Name** | **Length (aa)** | **Homologous P450 gene in *B. mori*** | **%identity** | **RNA-seq Contig ID** |
| --- | --- | --- | --- | --- |
| *CYP9A164* | 529 | *CYP9A19* | 56% | comp38522_c0, comp32566_c0, comp32566_c1 |
| *CYP9A165* | 530 | *CYP9A19* | 55% | comp1119_c0, comp33402_c0, comp1957_c0 |
| *CYP9A166** | 531 | *CYP9A20* | 62% | comp40281_c0 |
| *CYP9A167** | 535 | *CYP9A20* | 60% | comp42894_c6 |
| *CYP9A168** | 538 | *CYP9A20* | 56% | comp39193_c0 |
| *CYP9A169* | 486 | *CYP9A21* | 67% | comp37849_c0, comp37849_c1, comp37849_c2 |
| *CYP9A170** | 542 | *CYP9A21* | 62% | comp41752_c0, comp42752_c1 |
| *CYP9A171* | 560 | *CYP9A21* | 59% | comp39156_c0, comp39156_c2 |

Sequences of eight *CYP9A* genes of *A. honmai* were manually identified based on the sequences of RNA-seq contigs and genome scaffolds of *A. honmai* and the *CYP9A* genes of *B. mori*. % identity was calculated by blastp search. Accession IDs of *B. mori CYP9A* genes are ABO07439 (*CYP9A20*), ABP02071 (*CYP9A19*), and ABN71369 (*CYP9A21*). Asterisks indicate genes whose mRNA expression levels were analyzed by RT-qPCR.

**Table S8. *Adoxophyes honmai* strains used for RT-qPCR analysis.**

| **Strain number** | **Collection site** | **Prefecture** | **Latitude, Longitude** | **Collection year** |
| --- | --- | --- | --- | --- |
| 1 | Iwai, Bando | Ibaraki | N36.06, E139.87 | 2016 |
| 2 | Kamiyaganuki, Iruma | Saitama | N35.80, E139.34 | 2016 |
| 3 | Chirancho, Minamikyushu | Kagoshima | N31.28, E130.39 | 2015 |
| 4 | Harunocho, Hamamatsu | Shizuoka | N34.97, E137.95 | 2014 |
| 5 | Ishidera, Wazuka | Kyoto | N34.79, E135.88 | 2015 |
| 6 | Ishidera, Wazuka^a^ | Kyoto | N34.79, E135.88 | 2016 |
| 7 | Tsubaisocho, Kameyama | Mie | N34.87, E136.45 | 2016 |
| 8 | Ishidera, Wazuka^a^ | Kyoto | N34.79, E135.88 | 2016 |
| 9 | Higashihagima, Makinohara | Shizuoka | N34.77, E138.14 | 2016 |
| 10 | Nunohikihara, Makinohara | Shizuoka | N34.77, E138.15 | 2016 |
| 11 | Suizawacho, Yokkaichi | Mie | N34.86, E136.49 | 2016 |
| 12 | Yui, Shimada | Shizuoka | N34.81, E138.19 | 2015 |
| 13 | Kurasawa, Kikugawa^b^ | Shizuoka | N34.78, E138.14 | 2010 |
| ^a^ These larvae were collected from adjacent tea fields.  ^b^ Methoxyfenozide selected strain | | | | |

**Table S9. Correlations between the *CYP9A* gene expression and the corrected mortality in 13 *Adoxyophyes honmai* strains.**

| Gene name | *r** | *p*-value | n |
| --- | --- | --- | --- |
| *CYP9A166* | -0.4641 | 0.1101 | 13 |
| *CYP9A167* | -0.768 | 0.0022 | 13 |
| *CYP9A168* | -0.663 | 0.0135 | 13 |
| *CYP9A170* | -0.4088 | 0.1654 | 13 |
| Four *CYP9As* | -0.7845 | 0.0015 | 13 |

*Spearman rank correlation coefficient.

**Table S10. Correlations between the *CCE and GST* gene expression and the corrected mortality in 13 *Adoxyophyes honmai* strains.**

| Gene name | *r** | *p*-value | n |
| --- | --- | --- | --- |
| *AhCCE1* (comp32052_c0) | -0.3370 | 0.2601 | 13 |
| *AhCCE2* (comp37553_c0) | -0.6464 | 0.0170 | 13 |
| *AhCCE3* (comp37553_c3) | -0.6409 | 0.0183 | 13 |
| *AhCCE4* (comp40734_c2) | -0.895 | < 0.0001 | 13 |
| *AhCCE5* (comp41808_c0) | -0.4033 | 0.1718 | 13 |
| *AhCCE6* (comp44046_c0) | -0.5359 | 0.0591 | 13 |
| Six *AhCCEs* | -0.7624 | 0.0024 | 13 |
| *AhGST1* (comp38851_c0) | 0.2707 | 0.3710 | 13 |

*Spearman rank correlation coefficient.

**Table S11. Methoxyfenozide selection of *Adoxyophyes honmai*.**

| **Methoxyfenozide** | | |
| --- | --- | --- |
| **F Generation** | **Concentration** | |
|  | **Dilution rate** | **ppm** |
| 2 | 32000 | 3.13 |
| 3 | 32000 | 3.13 |
| 4 | NT | − |
| 5 | NT | − |
| 6 | 16000 | 6.25 |
| 7 | 12000 | 16.7 |
| 8 | 10000 | 12.5 |
| 9 | 10000 | 12.5 |
| 10 | 8000 | 25 |
| 11 | NT | − |
| 12 | 8000 | 25 |
| 13 | 8000 | 25 |
| 14 | 4000 | 50 |
| 15 | 4000 | 50 |
| 16 | 4000 | 50 |
| 17 | 2500 | 80 |
| 18 | NT | − |
| 19 | 2000 | 100 |
| 20 | NT | − |
| 21 | NT | − |
| 22 | 4000 | 50 |

NT: Generations not treated with Methoxyfenozide.

**Table S12. Primers used in each analysis.**

| **Primer name** | **Sequence (5’- 3’)** | **Analysis** |
| --- | --- | --- |
| AhEcR_Seq_F | GCTTGCTCAAGCGAGGTAAT | PCR/sequencing of *AhEcR* |
| AhEcR_Seq_R | TAGGAGCGCGTAATGGATGT | PCR/sequencing of *AhEcR* |
| AhEcR_Seq_F2 | TGACGCTATTGTATTGTGGTTTC | Sequencing of *AhEcR* |
| EcoRI_KOZAK_AhEcRB1_F | TTTGAATTCCCACCATGGATGTTTTTGAAGTTGGATCTGTGCGAAG | Cloning of *AhEcR* |
| XbaI_AhEcRB1_R | TTTTCTAGACTAGAGATTCATCGCGGACTCG | Cloning of *AhEcR* |
| EcoRI_KOZAK_AhUSP_F | TTTGAATTCCCACCATGGATGTCAAGTGTGGCGAAGAAAGACAAG | Cloning of *AhUSP* |
| XbaI_AhUSP_R | TTTTCTAGATTACATCATCGAGTTAACGTCGATCGG | Cloning of *AhUSP* |
| rp49_PCR_F | ATGGCAATCAGACCAGTTTACAGACC | Cloning of *rp49* |
| rp49_PCR_R | TTATTCGGCCTCCTGGCTCC | Cloning of *rp49* |
| comp40281_XbaI_F | TTTTCTAGAATGCTGTTCTACGTCTGGATAGC | Cloning of *CYP9A166* |
| comp40281_ApaI_R | TTTGGGCCCTCACTCCCGAGCCCTGAAC | Cloning of *CYP9A166* |
| comp42894_ XbaI_F | TTTTCTAGAATGATATTCTACATTTGGGCAGTG | Cloning of *CYP9A167* |
| comp42894_ApaI_R | TTTGGGCCCCTAATTCCTCGCTGTAAGATGG | Cloning of *CYP9A167* |
| comp39193_ XbaI_F | TTTTCTAGAATGATATTCGTGGTACTACTAGC | Cloning of *CYP9A168* |
| comp39193_ApaI_R | TTTGGGCCCTTATTTACGAGCCCGGAACC | Cloning of *CYP9A168* |
| comp41752_ XbaI_F | TTTTCTAGAATGCGGGCGACCATGAGT | Cloning of *CYP9A170* |
| comp41752_ApaI_R | TTTGGGCCCTTATTCTCTGATCTTCAGCCTTATCC | Cloning of *CYP9A170* |
| rp49_qPCR_F | CCGTCACATGCTACCCAATG | RT_qPCR of *rp49* |
| rp49_qPCR_R | CAGTAGGACCTGTTCTGCATC | RT_qPCR of *rp49* |
| comp40281_qPCR_F | CTTTGGGCTGAAAGTGGACTC | RT_qPCR of *CYP9A166* |
| comp40281_qPCR_R | CCTTCAAAAGTGCCAGGAAACC | RT_qPCR of *CYP9A166* |
| comp42894_qPCR_F | GGTCAGACACAGACTTGGTG | RT_qPCR of *CYP9A167* |
| comp42894_qPCR_R | CAGCTCGTAGAGGAAGAAGG | RT_qPCR of *CYP9A167* |
| comp39193_qPCR_F | TCCACAACCATGGCCTTCCT | RT_qPCR of *CYP9A168* |
| comp39193_qPCR_R | CGTTGTTCCGCTCCTCGTTT | RT_qPCR of *CYP9A168* |
| comp41752_qPCR_F | GGCACAGGAGATCAGGGAAA | RT_qPCR of *CYP9A170* |
| comp41752_qPCR_R | CTCGGAGACAACCATGTCCA | RT_qPCR of *CYP9A170* |
| comp38851_qPCR_F | CAACACCCTGTACGCCAGAT | RT_qPCR of *AhGST1* |
| comp38851_qPCR_R | CTCCAGGAATCCAACGGCTT | RT_qPCR of *AhGST1* |
| comp32052_qPCR_F | GCTCCTCAACCTCCAACTCC | RT_qPCR of *AhCCE1* |
| comp32052_qPCR_R | GTAGAGGCAGTCTTCGCTCC | RT_qPCR of *AhCCE1* |
| comp37553_c0_qPCR_F | GTCCACATGCCTGACACAGA | RT_qPCR of *AhCCE2* |
| comp37553_c0_qPCR_R | AAGGATGGGGTGCCATGATG | RT_qPCR of *AhCCE2* |
| comp37553_c3_qPCR_F | GCCGCTGGGAGTGATTCTAT | RT_qPCR of *AhCCE3* |
| comp37553_c3_qPCR_R | TGACTGCGCGCAATGATTTGC | RT_qPCR of *AhCCE3* |
| comp40734_qPCR_F | CAATGCGGGCATGAAAGACC | RT_qPCR of *AhCCE4* |
| comp40734_qPCR_R | CTGAGGATCCAGCGCTAGT | RT_qPCR of *AhCCE4* |
| comp41808_qPCR_F | ACAAGACCGAAGACGACGTG | RT_qPCR of *AhCCE5* |
| comp41808_qPCR_R | CATCCAAACCGGTCCAGGTA | RT_qPCR of *AhCCE5* |
| comp44046_qPCR_F | GGAGGAGGCTTCTTCAGTG | RT_qPCR of *AhCCE6* |
| comp44046_qPCR_R | AACCGAGGACATCAAGCCTG | RT_qPCR of *AhCCE6* |

Restriction enzyme sites are boxed. The Kozak sequence is underlined

**Supplementary Methods**

**Bioassays.** Corrected mortality (%) of the strains used for RT-qPCR analysis (Table S8) was determined by leaf dipping method described previously^S1^. Briefly, tea leaves were dipped with 200 ppm of tebufenozide (Nihon Nohyaku) dissolved in water containing 0.01% Tween 20. Control leaves were treated with water containing only 0.01% Tween 20. Ten 2nd or 3rd instar larvae were placed on six treated leaves in a plastic dish and mortality was examined after 10 days. Each assay was performed in triplicate. Corrected mortality (%) was calculated using Abott’s formula as follows:100 x (survival rate in the control - survival rate in the test) / survival rate in the control^S2^.

**Analysis of DEGs based on RNA-seq.** RNA-seq data obtained from two tebufenozide-resistant strains (Yui2014-R and Met-Sel-R) and two tebufenozide-susceptible strains (Kanaya1960-S and Haruno2014-S) were used for the analysis of DEGs. Total RNA was extracted from one-day-old third-instar individuals of each strain without exposing to any insecticides, using the RNeasy Plus Mini kit (QIAGEN). Three replicates of total RNA samples (one individual for each replicate) were prepared for each strain. A cDNA library was constructed for each RNA sample and sequenced on the Illumina HiSeq 2000 platform using the 101-bp paired-end mode by Macrogen Japan Corp. The obtained reads were cleaned using Trimmomatic 0.32^S3^. The cleaned reads were mapped onto the reference transcriptome assembly, and the expression level of each gene was calculated using the “align_and_estimate_abundance.pl” program bundled with Trinity^S4^. Trinity generates clusters of transcript sequences and assigns a gene ID to each cluster. We used the Trinity genes in the reference transcriptome assembly for calculating gene level expression. Analysis of DEGs between each pair of susceptible and resistant strains was performed through the iDEGES/edgeR method^S5^ with false discovery rate (FDR) < 0.05, using the gene level expression data of three replicates for each strain. DEGs of each resistant strain were narrowed down by extracting common DEGs found for both the susceptible strains, respectively. The common DEGs were further narrowed down by extracting common DEG found in both the resistant strains.

**Identification of corresponding *B. mori* chromosomes for *A. honmai* linkage groups (LGs).** Each RAD locus mapped on the LGs was compared with the draft *A. honmai* genome assembly by blastn search (identity > 97% and query coverage > 90%) and top hit scaffolds were extracted. Each top hit scaffold was compared with *B. mori* genome sequences in KAIKObase (http://sgp.dna.affrc.go.jp/KAIKObase/) by blastn search (e-value < 1e-10) and a top hit *B. mori* chromosome was identified based on the highest total score of High Scoring Pairs (HSPs) (only one highest HSP was counted for alignments overlapped in the query region). Similarly, top hit RNA-seq contigs in the representative transcriptome assembly were also extracted by blastn search (identity > 97% and e-value < 1e-20) for each RAD locus. Each top hit RNA-seq contig was compared with gene set A of *B. mori* in KAIKObase by blastx search (e-value < 1e-5) and a top hit *B. mori* chromosome was identified based on the top hit *B. mori* gene. Corresponding *B. mori* chromosome of each *A. honmai* LG was estimated based on the total number of top hit scaffolds and RNA-seq contigs mapped on each *B. mori* chromosome.

**Identification of candidate loci responsible for tebufenozide resistance by ddRAD-seq analysis.** A chi-squared test was performed for all SNP markers extracted by the ddRAD-seq analysis to evaluate deviations in the genotype frequencies of F_2_ survivors from the Mendelian expected ratio (1:2:1). Because tebufenozide resistance in *A. honmai* is inherited as an autosomal and incompletely dominant trait^S6^, SNP markers with a Bonferroni-corrected p-value < 0.05/N (N = unique SNP markers on each LG) and high genotype frequency (> 90%) of resistant homozygous (R/R) or heterozygous (R/S) were searched to identify candidate genomic regions for tebufenozide resistance. Synteny analysis between the candidate regions of *A. honmai* and *B. mori* genome^S7^ was then performed. Each SNP marker in the candidate regions was mapped to a genome scaffold of *A. honmai* by blastn search and *B. mori* genes^S8^ were mapped to the target *A. honmai* scaffold by tblastn search. Conserved synteny between the two insect genomes was identified based on the order of *B. mori* genes mapped to the target scaffolds and used for elucidating the candidate loci responsible for tebufenozide resistance.

**Determination of *AhEcR* A415V genotypes in ddRAD samples.** A DNA fragment (190 bp) of *EcR* in *A. honmai* encoding a peptide encompassing A415V in AhEcRB1 (exon 6 of the B1 isoform) was amplified by PCR and Sanger sequenced. The PCR mixture (15 μl) contained 1× Ex Taq buffer, dNTPs (0.2 μM each), AhEcR_Seq_F and AhEcR_Seq_R primers (1 μM each; Table S10), and genomic DNA (1 μl ddRAD-seq samples 100-fold diluted with distilled water), and Ex Taq (0.375 U; TaKaRa Bio). Amplification conditions were as follows: 94 °C for 2 min; five cycles at 94 °C for 20 s, 65 °C for 30 s, and 72 °C for 1 min; five cycles at 94 °C for 20 s, 60 °C for 30 s, and 72 °C for 1 min; 35 cycles at 94 °C for 20 s, 55 °C for 30 s, and 72 °C for 1 min. After PCR, the reaction mixture (3 μl) were mixed with Illustra ExoProStar (2 μl; GE Healthcare) to prepare template DNA ready for direct-sequencing. Sequencing was performed in a total reaction mixture (10 μl) containing the template DNA (5 μl), 1× Sequencing Buffer, AhEcR_Seq_F2 primer (3.6 μM), and BigDye Terminator v3.1 Ready Reaction Mix (1 μl; Thermo Fisher Scientific). The conditions for sequencing reaction were as follows: 96 °C for 1 min, followed by 25 cycles 96 °C for 10 s, 55 °C for 5 s, and 60 °C for 4 min. DNA sequencing performed using the ABI PRISM 3100 Genetic Analyzer (Applied Biosystems).

**Cloning and sequencing of *AcEcRB1* cDNAs and construction of expression plasmids.** Total RNAs were extracted from third instar larvae of Kanaya1960-S and Yui2014-R strains using the RNeasy Plus Mini kit (QIAGEN), and cDNAs were synthesized from the total RNAs (500 ng) using the PrimeScript RT reagent kit (TaKaRa Bio). Full open reading frames (ORFs) of the *EcR isoform B1* of the susceptible strain (*AhEcRB1_S*) and the resistant strain (*AhEcRB1_R*) were amplified from the respective cDNA. The full ORFs of *Ultraspiracle isoform 1* of *A. honmai* (*AhUSP-1*) were amplified by PCR from the cDNAs of Kanaya1960-S. The PCRs were performed in total reaction mixture (50 μl) containing 1× PCR Buffer for KOD-Plus-Neo, dNTPs (0.2 mM each), MgSO_4_ (1.5 mM), forward and reverse primers (0.3 μM), cDNA template (1 μl), and KOD-Plus-Neo polymerase (1 U; TOYOBO). Primers used for *AhEcRB1* (EcoRI_KOZAK_AhEcRB1_F/XbaI_AhEcRB1_R) and *AhUSP1* (EcoRI_KOZAK_AhUSP_F/XbaI_AhUSP_R) are described in Table S12. The PCR conditions were as follows: 94 °C for 2 min; five cycles at 98 °C for 10 s and 70 °C for 1 min; five cycles at 98 °C for 10 s, 65 °C for 30 s, and 68 °C 1 min; 35 cycles at 98 °C for 10 s, 59 °C for 30 s, and 68 °C for 1 min. The amplicons were separated by 1% agarose gel electrophoresis, and then purified with the Wizard SV Gel and PCR Clean-Up System, digested with restriction enzymes (XbaI and EcoRI), and subcloned into the pTnT vector (all Promega), according to the manufacture’s protocols. Plasmid sequences were verified by sequencing using the BigDye Terminator v3.1 Cycle Sequencing Kit (Thermo Fisher Scientific) and the PCR primers mentioned above, under the conditions described in the “Determination of *AhEcR* A415V genotypes in ddRAD samples” section.

**RNA extraction and cDNA preparation for RT-qPCR.** Living *A. honmai* larvae (4th or 5th instar) were immediately homogenized individually in a 1.5 ml tube containing 350 µl RLT Plus buffer plus 10 mM β-mercaptoethanol (QIAGEN) using a plastic pestle. Total RNA was extracted from the homogenate using the RNeasy Plus Mini Kit (QIAGEN) following manufacture’s instruction. Genome DNA was eliminated with a gDNA Eliminator spin column (QIAGEN). Three larvae were used for RNA extraction in each strain listed in Supplementary Table S8. Concentration and integrity (A_260/_A_280_ ratio) of the extracted RNA were examined using NanoDrop ND-1000 (Thermo Fisher Scientific). Template cDNAs were synthesized using the Prime Script RT reagent Kit (Takara Bio) following manufacture’s instruction. The extracted RNA (500 ng) was reverse-transcribed in a 10 µl reaction mixture containing 1 x PrimeScript Buffer, PrimeScript Enzyme RT Mix I (0.5 μl), oligo dT primer (25 pmol), and random 6 mers (50 pmol) at 37 ^o^C for 15 min, and the reaction was stopped by heating at 85 ^o^C for 5 sec.

**RT-qPCR for *P450* genes.**  Absolute quantification was performed for four target *P450* genes (*CYP9A166*, *CYP9A167*, *CYP9A168*, and *CYP9A170*) and *rp49* using LightCycler 480 (Roche Applied Science). In a preliminary experiment, we verified that the expression stability of three house-keeping genes (*rp49*, *EF-1α*, and *β-actin*) were nearly equal in *A. honmai* cDNA samples, and we selected *rp49* as a reference gene throughout this study. Standard DNAs for the *P450s* and *rp49* were amplified by PCR from the cDNA of the Yui2014-R strain using the cloning primers (Supplementary Table S12) and subcloned into the pGEM-T plasmid (Promega). The qPCR was carried out in a reaction mixture (10 µl) containing *A. honmai* cDNA (equivalent to 5 ng total RNA) or the standard plasmid DNA of *P450*s and *rp49* (1 ng to 10^-6^ ng, 10-fold dilution series), 1× SYBR Premix Ex Taq II (Takara Bio), and a pair of target gene-specific qRT-PCR primers (0.2 µM each) (Supplementary Table S12). PCR conditions were as follows: 95 °C for 30 s followed by 50 cycles at 95 °C for 5 s and 65 °C for 20 s. After PCR, the specificity of PCR was confirmed by the automated melting curve analysis. The absolute amounts of the *P450* and *rp49* transcripts were calculated using respective standard curves obtained by the Second Derivative Maximum method (LightCycler 480 software version 1.5.1.62.SP3). The PCR efficiencies calculated from the slope of standards were as follows: *rp49*, 1.01; *CYP9A166*, 1.01; *CYP9A167*, 0.89; *CYP9A168*, 0.98; and *CYP9A170*, 0.86. The expression level of *P450* genes in each sample was normalized to that of *rp49* gene, and the average and standard deviation were calculated from three biological replicates in each strain by Microsoft Excel. Spearman rank correlation coefficient between the average expression level and the corrected mortality in each strain was calculated by GraphPad PRISM Version 4 (GraphPad Software).

**RT-qPCR for *CCE* and *GST* genes.** Relative quantification was performed for *AhCCE1-6* and *AhGST1* genes, using LightCycler 96 (Roche Applied Science). The above-mentioned cDNA samples were analyzed. *rp49* was selected for a reference gene. The qPCR was carried out in a reaction mixture (10 µl) containing a cDNA sample (equivalent to 5 ng total RNA), 1× SYBR Premix Ex Taq II (Takara Bio), and a pair of target gene-specific qRT-PCR primers (0.2 µM each) (Supplementary Table S12). PCR conditions were as follows: 95 °C for 30 s followed by 50 cycles at 95 °C for 5 s and 65 °C for 20 s. Each sample was analyzed both for target genes and *rp49*. After PCR, the specificity of PCR was confirmed by the automated melting curve analysis. Standard curves were prepared from 6 points of 10-fold dilution series of a cDNA sample of Yui2015-R strain. The PCR efficiencies were determined from the slope of standard curves as follow: *rp49*, 1.01; *AhCCE1*, 1.06; *AhCCE2*, 1.05; *AhCCE3*, 1.01; *AhCCE4*, 1.09; *AhCCE5*, 0.81; *AhCCE6*, 0.83; and *AhGST1*, 1.14. The expression level of target genes normalized to that of *rp49* was calculated based on the comparative C_T_ method ^S11^ using LightCycler 96 Software version 1.1.0.1320 (Roche Applied Science) and the average and standard error were calculated from three biological replicates in each strain by Microsoft Excel. Spearman rank correlation coefficient between the average expression level and the corrected mortality in each strain was calculated by GraphPad PRISM Version 4 (GraphPad Software).

**Supplementary References**

1. Uchiyama, T. & Ozawa, A. Rapid development of resistance to diamide insecticides

in the smaller tea tortrix, *Adoxophyes honmai* (Lepidoptera: Tortricidae), in the tea fields of Shizuoka Prefecture, Japan. *Appl Ent Zool* **49**, 529-534 (2014).

2. Abbott, W. S. A method of computing the effectiveness of an insecticide. *J Eco Entomol* **18**, 265-267 (1925).

3. Bolger, A. M., Lohse, M. & Usadel, B. Trimmomatic: a flexible trimmer for Illumina sequence data. *Bioinformatics* **30**, 2114–2120 (2014).

4. Haas, B. J. *et al*. De novo transcript sequence reconstruction from RNA-Seq: reference generation and analysis with Trinity. *Nat Protoc* **8**, (2013).

5. Sun, J., Nishiyama, T., Shimizu, K. & Kadota, K. TCC: An R package for comparing tag count data with robust normalization strategies. *BMC Bioinformatics* **14,** 219 (2013).

6. Uchiyama, T. & Ozawa, A. Inheritance of tebufenozide resistance in the smaller tea tortrix, *Adoxophyes honmai* (Lepidoptera: Tortricidae). *Jpn J Appl Ent Zool* **59**, 127-131 (2015).

7. Xia, Q. *et al*. The genome of a lepidopteran model insect, the silkworm *Bombyx mori*. *Insect Biochem. Mol. Biol.* **38**, 1036-1045 (2008).

8. Suetsugu, Y *et al.* Large scale full-length cDNA sequencing reveals a unique genomic landscape in a lepidopteran model insect, *Bombyx mori*. G3 (Bethesda) **3,** 1481-1492.

9. Billas, I. M. *et al.* Structural adaptability in the ligand-binding pocket of the ecdysone hormone receptor. *Nature* **426**, 91-96 (2003).

10. Nakagawa, Y. *et al.* Molecular cloning of the ecdysone receptor and the retinoid X receptor from the scorpion *Liocheles australasiae*. *FEBS J* **274**, 6191-6203 (2007).

11. Schmittgen, T. D. & Livak, K. J. Analyzing real-time PCR data by the comparative C_T_ method. *Nat Protoc* **3**, 1101-1108 (2008).

**Supplementary figures**

**
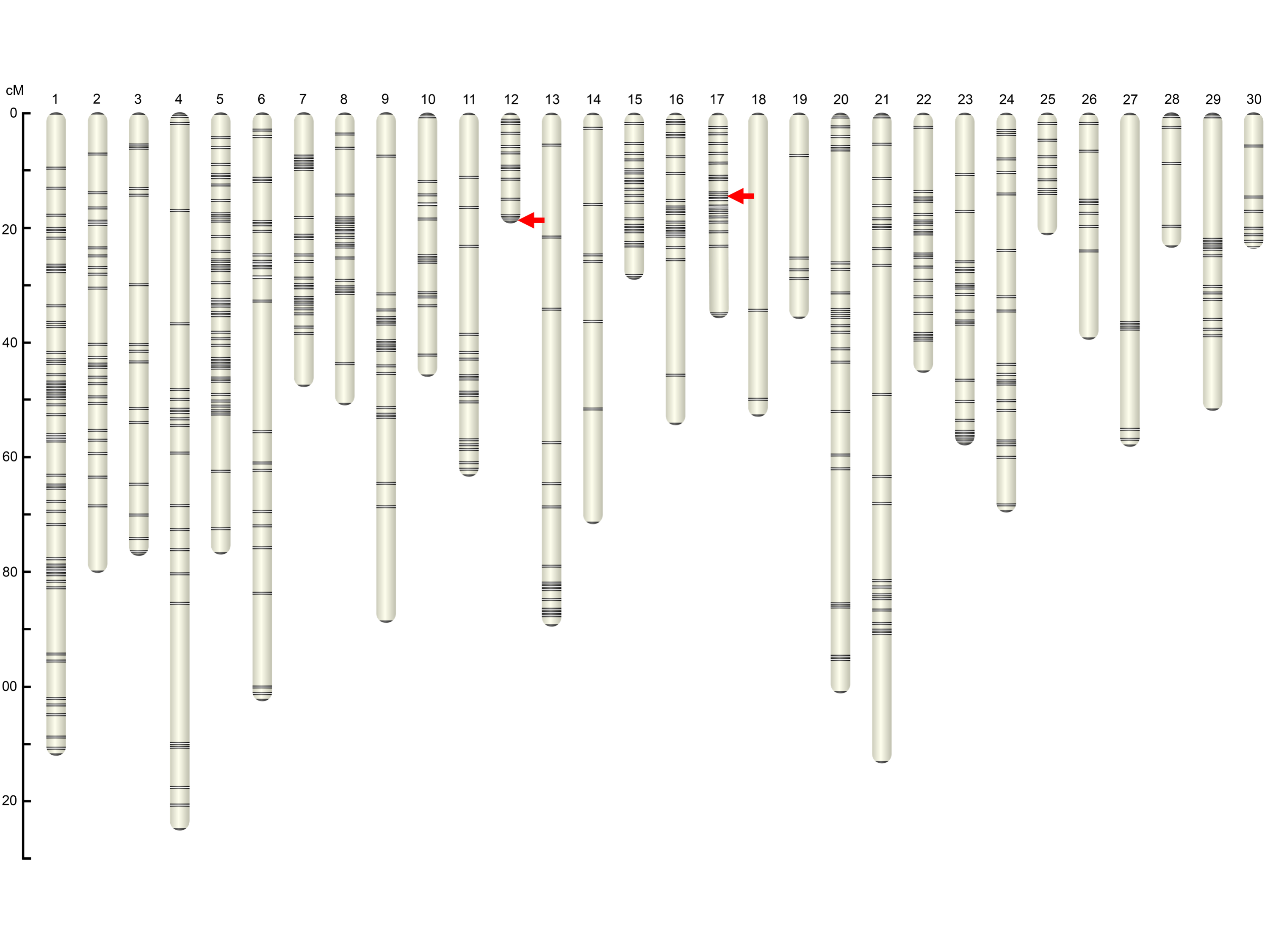
Figure S1. Linkage map of *Adoxophyes honmai*.** The linkage map was generated by JoinMap using the 945 single nuclear polymorphism markers obtained from double-digest restriction site-associated DNA sequencing data. The linkage map consists of 30 linkage groups with 927 SNP markers on 579 unique positions indicated with black lines. The details of the SNP markers are described in Table S5. Arrows indicates candidate loci responsible for tebufenozide resistance identified by ddRAD-seq analysis. See more detail in the text.

**
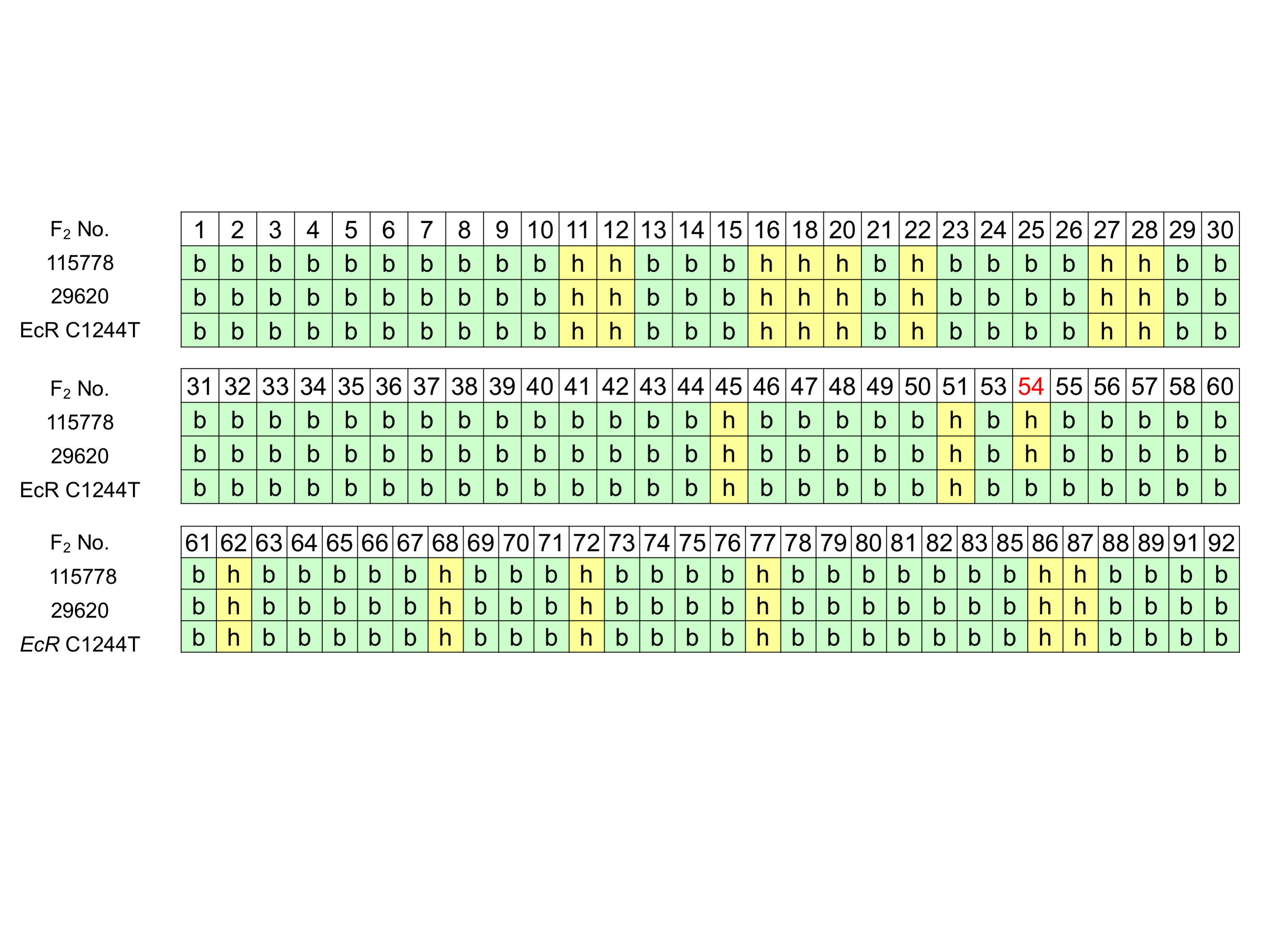
Figure S2. Comparison of SNP markers on LG17 and SNP C1244T in *EcR* in *Adoxyohyes honmai*.** The genotypes of the SNP C1244T, which corresponds to A415V substitution in AhEcRB1, were determined individually in 89 F2 progenies used for ddRAD-seq analysis. The SNP markers 115778 and 29620 were identified as significant markers for tebufenozide resistance by the ddRAD-seq analysis. ”b” and “h” indicate “R/R” and “R/S” genotypes, respectively. No “S/S” genotype was found. Only one individual (No. 54 in red) showed the difference.


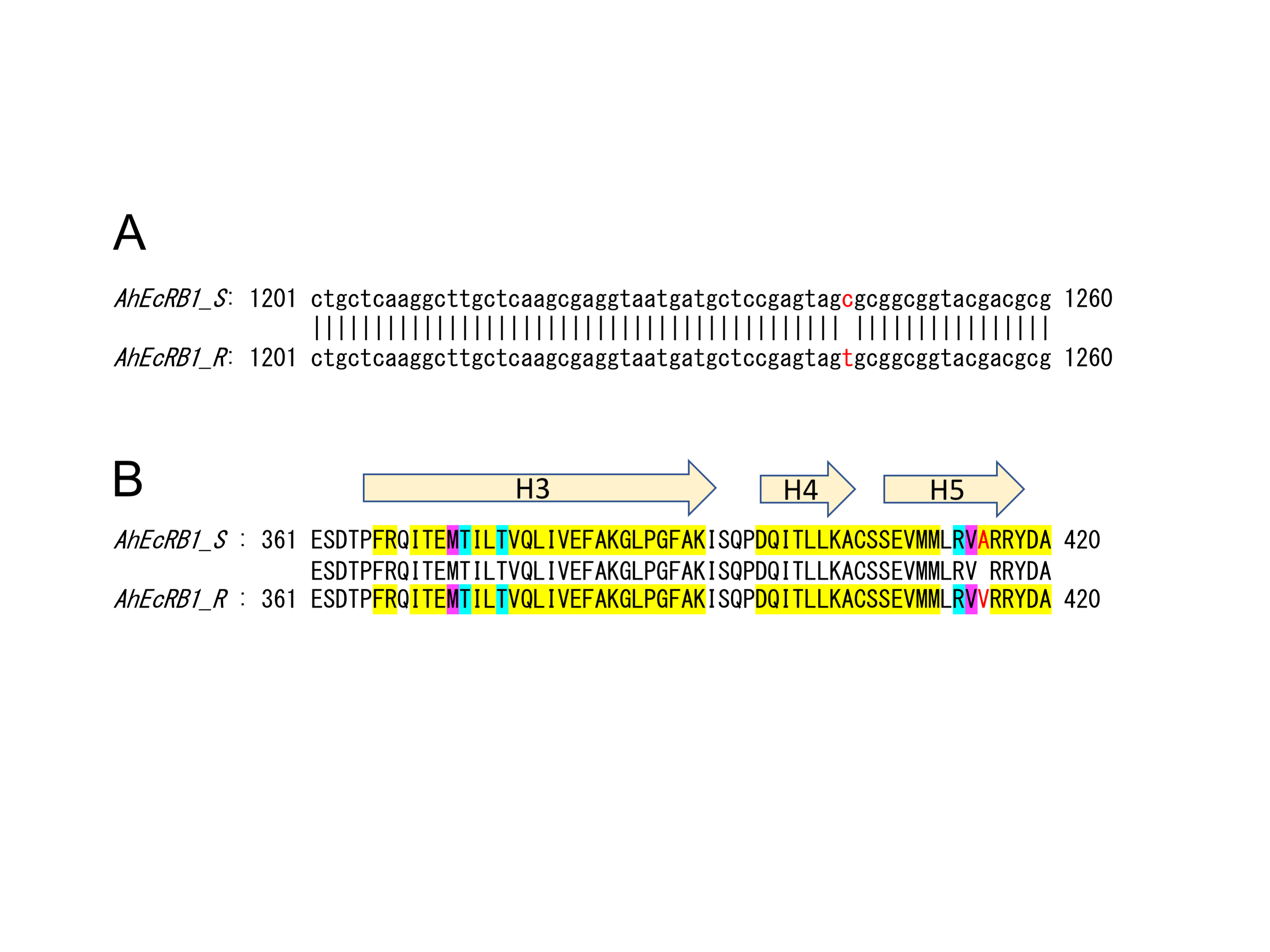


**Figure S3. Alignment of nucleotide (A) and deduced amino acid sequence (B) of *AhEcRB1_*S and *AhEcRB1_R*.** Red characters indicate different nucleotides (A) and amino acid residues (B). Arrows: alfa-helixes, Blue: amino acid residues forming hydrogen-bond with ponasterone A^S9^, Yellow: conserved amino acid residues among insects, Magenta: amino acid residues specifically conserved in Lepidoptera^S10^.


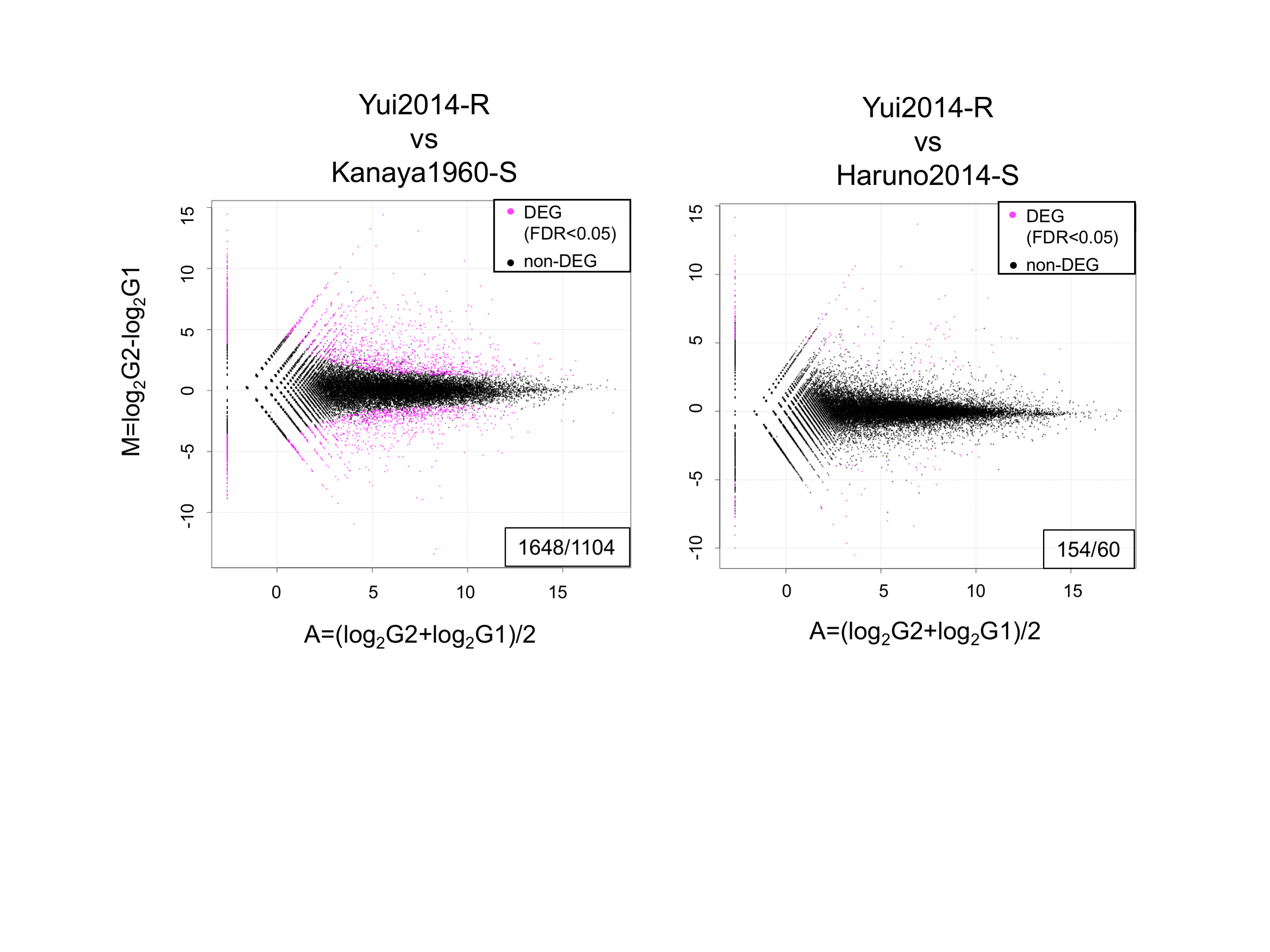


**Figure S4. MA plots of DEG analysis between the tebufenozide resistant strain (Yui2014-R) and susceptible strains (Kayana1960-S and Haruno2014-S).** X-axis and y-axis show A (log average expression) and M (log fold change) numbers, respectively. Number in the bottom-right box indicates number of up-regulated DEGs/down –regulated DEGs in the resistant strain. Three individuals were sequenced for each strain in each comparison.

**
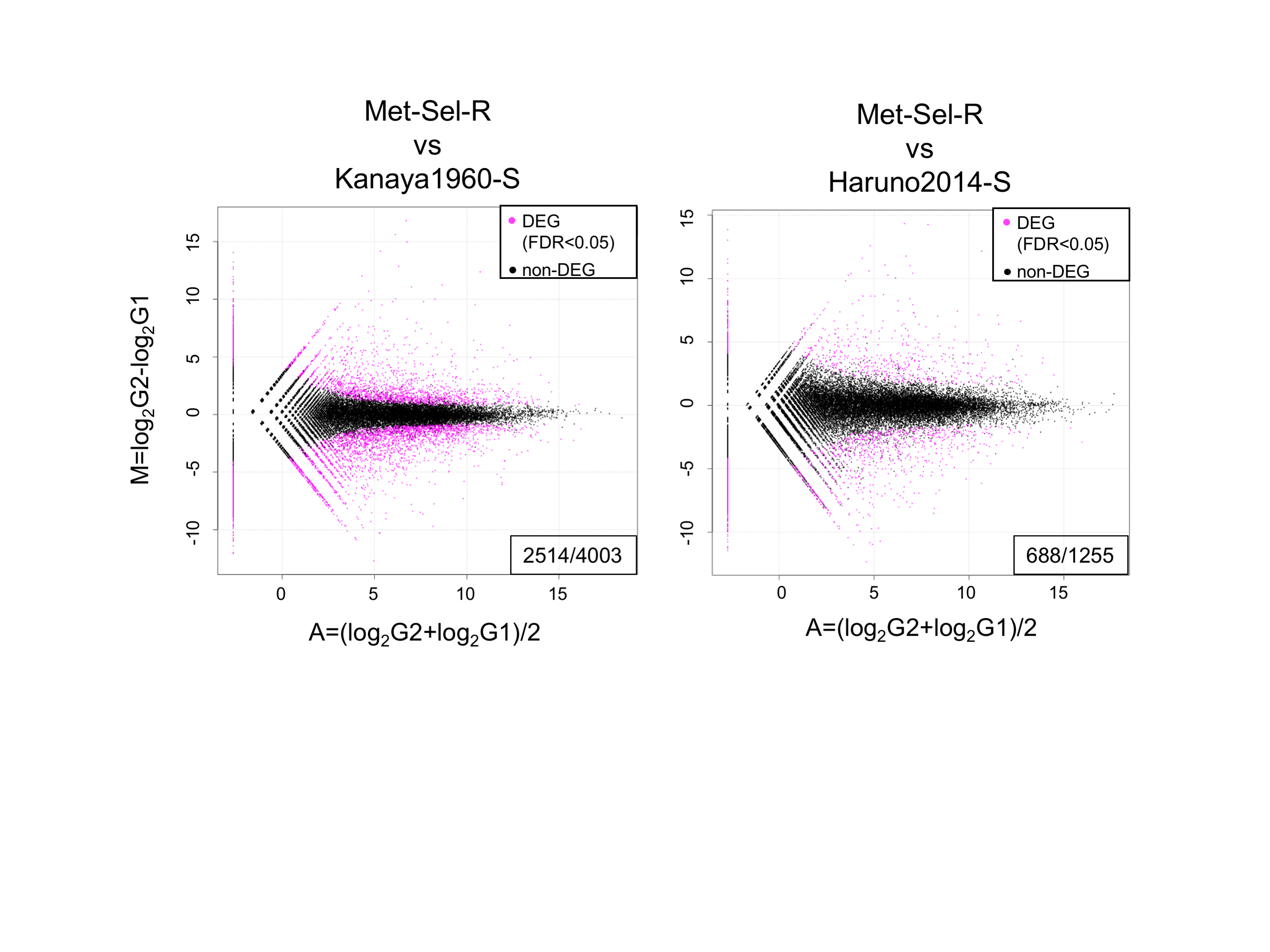
Figure S5. MA plots of DEG analysis between the methoxyfenozide resistant strain (Met-Sel-R) and susceptible strains (Kanaya1960-S or Haruno2014-S).** Detailed descriptions in the figure are the same as described in Fig. S4. Met-Sel-R is also resistant to tebufenozide. Number in the bottom right box indicates the number of up DEGs/down DEGs in R strain. Each strain used three individuals.

**
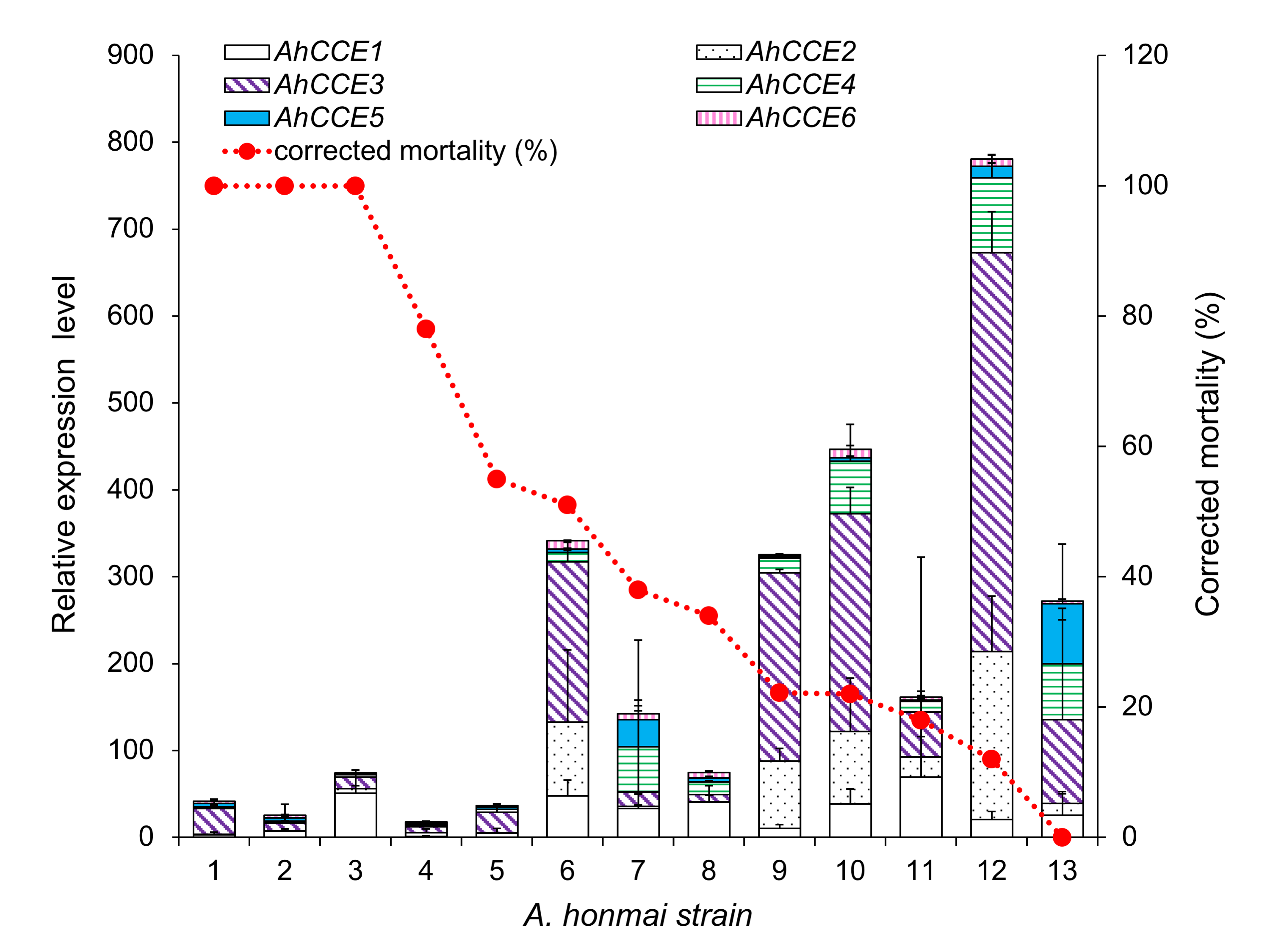
Figure S6. Relationship between the expression levels of six *choline/carboxylesterase* (*CCE*) genes and corrected mortality in 13 *Adoxophyes honmai* strains.** Bars indicate the expression levels of six *CCE* genes relative to a reference gene (*rp49*) as obtained by RT-qPCR (mean + standard error, n=3). The relative expression levels of *AhCCE1* (open bars), *AhCCE2* (dotted bars), *AhCCE3* (diagonal-striped bars), *AhCCE4* (horizontal-striped bars), *AhCCE5* (filled bars), and *AhCCE6* (vertical-striped bars) are indicated for each strain, along with corrected mortality (red dots). The numbers at the bottom indicate the strains of *A. honmai* listed in Supplementary Table S8.

**
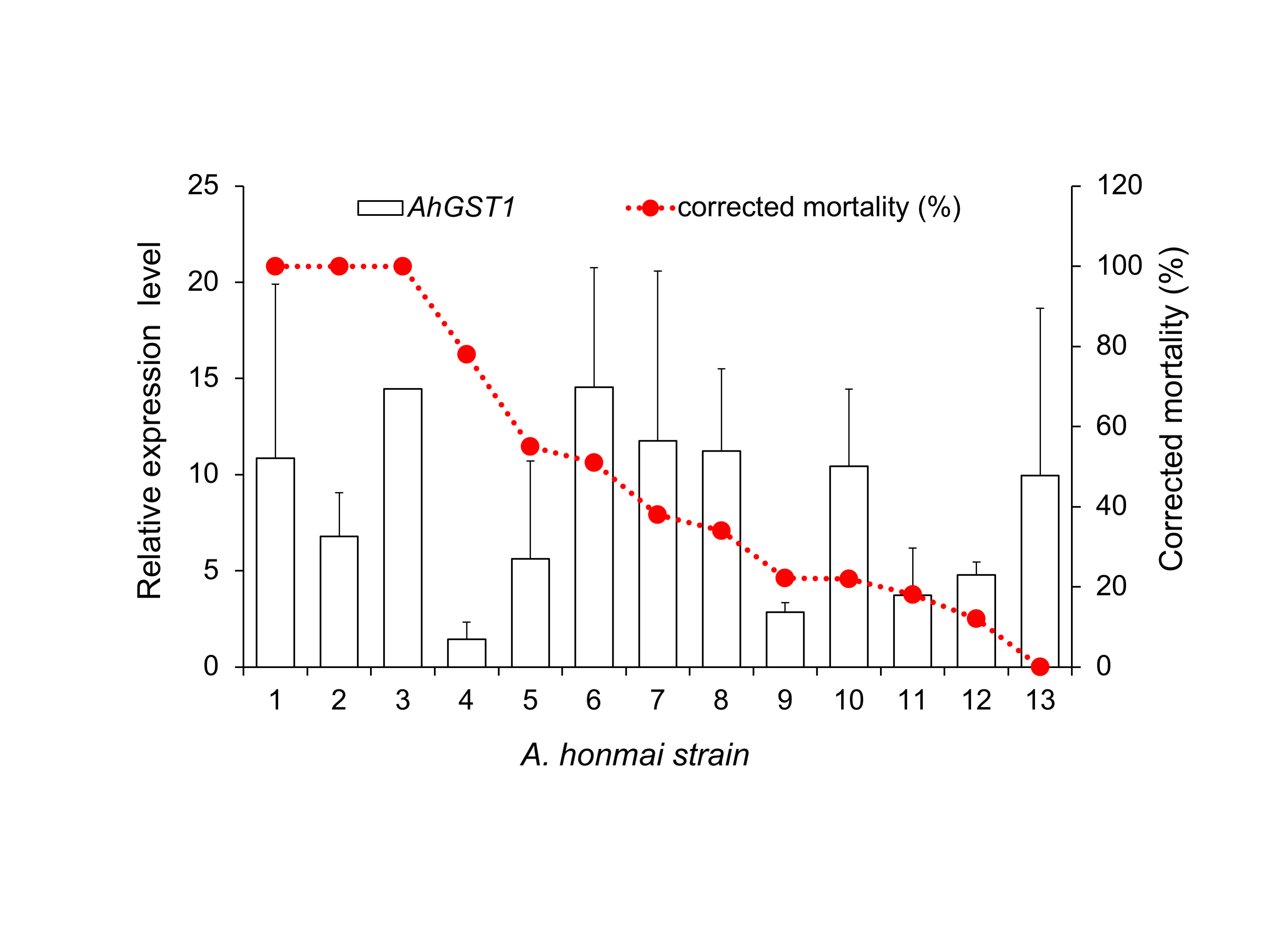
Figure S7. Relationship between the expression levels of a *glutathione S-transferase* (*GST*) gene and corrected mortality in 13 *Adoxophyes honmai* strains.** Bars indicate the expression levels of *AhGST1* relative to a reference gene (*rp49*) as obtained by RT-qPCR (mean + standard error, n=3). The relative expression level of *GST* is indicated for each strain, along with corrected mortality (red dots). The numbers at the bottom indicate the strains of *A. honmai* listed in Supplementary Table S8.
